# Transcriptome Changes Associated with Anaerobic Growth in *Yersinia intermedia* (ATCC29909)

**DOI:** 10.1371/journal.pone.0076567

**Published:** 2013-10-07

**Authors:** Lavanya Babujee, Venkatesh Balakrishnan, Patricia J. Kiley, Jeremy D. Glasner, Nicole T. Perna

**Affiliations:** 1 Biotechnology Center, University of Wisconsin - Madison, Madison, Wisconsin, United States of America; 2 Department of Biomolecular Chemistry, University of Wisconsin - Madison, Madison, Wisconsin, United States of America; 3 Department of Genetics, University of Wisconsin - Madison, Madison, Wisconsin, United States of America; Universidade Nova de Lisboa, Portugal

## Abstract

**Background:**

The yersiniae (Enterobacteriaceae) occupy a variety of niches, including some in human and flea hosts. Metabolic adaptations of the yersiniae, which contribute to their success in these specialized environments, remain largely unknown. We report results of an investigation of the transcriptome under aerobic and anaerobic conditions for *Y. intermedia*, a non-pathogenic member of the genus that has been used as a research surrogate for *Y. pestis*. *Y. intermedia* shares characteristics of pathogenic yersiniae, but is not known to cause disease in humans. Oxygen restriction is an important environmental stimulus experienced by many bacteria during their life-cycles and greatly influences their survival in specific environments. How oxygen availability affects physiology in the yersiniae is of importance in their life cycles but has not been extensively characterized.

**Methodology/Principal Findings:**

Tiled oligonucleotide arrays based on a draft genome sequence of *Y. intermedia* were used in transcript profiling experiments to identify genes that change expression in response to oxygen availability during growth in minimal media with glucose. The expression of more than 400 genes, constituting about 10% of the genome, was significantly altered due to oxygen-limitation in early log phase under these conditions. Broad functional categorization indicated that, in addition to genes involved in central metabolism, genes involved in adaptation to stress and genes likely involved with host interactions were affected by oxygen-availability. Notable among these, were genes encoding functions for motility, chemotaxis and biosynthesis of cobalamin, which were up-regulated and those for iron/heme utilization, methionine metabolism and urease, which were down-regulated.

**Conclusions/Significance:**

This is the first transcriptome analysis of a non-pathogenic *Yersinia* *spp.* and one of few elucidating the global response to oxygen limitation for any of the yersiniae. Thus this study lays the foundation for further experimental characterization of oxygen-responsive genes and pathways in this ecologically diverse genus.

## Introduction

*Yersinia intermedia* is a Gram-negative, motile, rod-shaped bacterium that belongs to the family Enterobacteriaceae. The ecologically diverse genus *Yersinia* includes 14 species [1], three of which, namely *Y. pestis*, *Y. pseudotuberculosis* and *Y. enterocolitica*, are human pathogens. The non-pathogenic species were grouped traditionally as “*Y. enterocolitica*-like” mainly to distinguish them from *Y. pseudotuberculosis* and *Y. pestis* [2]. All of the human-pathogenic yersiniae are descendants of a non-pathogenic predecessor according to a simplified evolutionary model [3]. Population genetics studies suggest that *Y. pseudotuberculosis* and *Y. enterocolitica* lineages diverged from a common ancestor somewhere between 150 and 200 million years ago and that *Y. pestis* was clonally derived from *Y. pseudotuberculosis* as recently as 1,500-20,000 years ago [4]. In support of these biochemical and genetic studies, phylogenetic analysis based on genome sequence data has identified three major clades among the yersiniae: *Y. pestis* /*Y. pseudotuberculosis*, the 'enterocolitica-like' species and the outlying fish pathogen, *Y. ruckeri* [5].

*Y. intermedia* was defined as a new species distinct from *Y. enterocolitica* in 1980 based on DNA-DNA hybridizations and biochemical characteristics [6]. The lipopolysaccharides (O-antigens) of *Y. intermedia* are similar to those of *Y. enterocolitica*, and its carbohydrate utilization profile is similar to that of *Y. enterocolitica* and *Y. pseudotuberculosis* [6]. *Y. intermedia* has been isolated mostly from aquatic environments such as freshwater and sewage as well as from invertebrate hosts (fish, oysters, shrimps, snails) living in aquatic environments. Association with mammals is evident from its isolation from sources such as milk, cream and meat. It has also been reported from human urine and stool specimens as well as from wound infections [7,8], but has only been rarely associated with disease in humans [9].

The type strain *Y. intermedia* ATCC29909 was isolated from a human urine sample and has been classified as biotype 1 and serogroup O17 [6]. The genome of *Y. intermedia* ATCC29909 was sequenced by the Naval Medical Research Center, USA [5]. The genome of this non-pathogenic strain differs from the pathogenic strains in key aspects. For example, unlike the pathogenic strains, the plasmid in *Y. intermedia* [10] does not encode genes for the type 3 secretion system apparatus (*ysc* -genes) and effectors (*yop* -genes). *Y. intermedia* also lacks genes for the synthesis of the siderophore yersiniabactin, which are located in a “high pathogenicity island” (HPI) on the chromosome of the enteropathogenic species [11].

While temperature and iron availability are considered the most important environmental factors globally governing expression of genes including virulence factors in the yersiniae [12,13], recent studies indicate that pH, oxygen-limitation, osmolarity and nutrient availability also modulate gene expression in *Y. pestis* [14]. For example, it has been shown that oxygen status influences expression of adhesins and genes required for attachment in the enteropathogenic yersiniae [15,16]. In fact, all three human-pathogenic yersiniae encounter oxygen limitation within natural niches, such as the human ileum or subcutaneous tissue, or the flea proventriculus/mid-gut [16,17,18]. There are limited studies on the effects of oxygen-availability on gene expression in the yersiniae, and even these studies are restricted to pathogenic species.

Genome sequences are available for at least one representative species for more than 20 different genera of enterobacteria (http://img.jgi.doe.gov/). To our knowledge changes in global gene expression under oxygen limitation have been documented for only five of them (*E. coli K-12 MG1655* [19]: *Salmonella enterica* serovar Typhimurium [20]:, *Shigella* [21]:, *Dickeya dadantii 3937* and *Pectobacterium atrosepticum SCRI1043* [22]:). Thus the range of metabolic responses to O2 and the mechanisms that govern them are still incompletely understood for this family of facultative anaerobes.

Here we describe results of our microarray-based transcriptome analysis of *Yersinia intermedia* ATCC29909 grown to early exponential phase with glucose as the sole carbon source with and without oxygen. We believe that robust experiment-based comparative transcriptomics studies such as this are important steps towards furthering our understanding of global regulatory mechanisms operating in the yersiniae. In addition such studies should prove useful for inferring patterns of regulatory evolution among the yersiniae as well as among the enterobacteria. Together with our existing knowledge of genetics and biochemistry, such approaches have the potential to help identify novel therapeutic agents and control strategies to combat devastating diseases caused by enterobacteria.

## Results and Discussion

### Transcriptome Profiling

**Figure 1:**
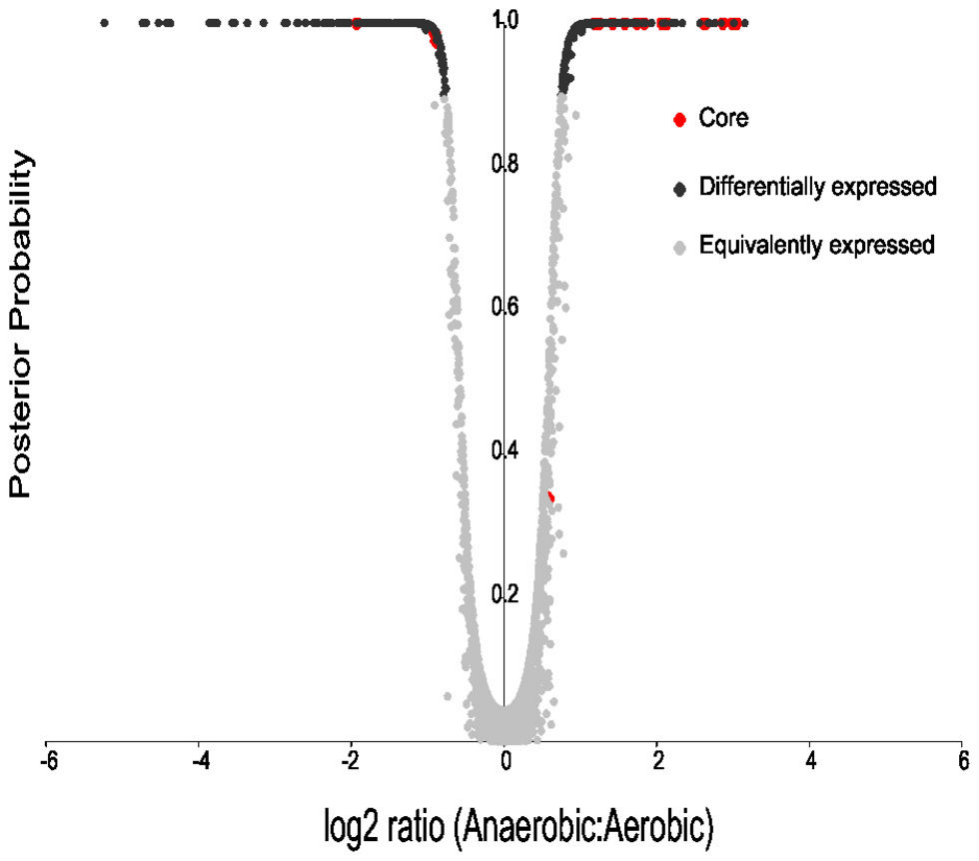
Volcano Plot of fold change versus significance. Gene expression data for *Y*. *intermedia* grown under aerobic and anaerobic conditions was used to derive log_2_ ratios (X-axis) which are plotted against posterior probability of differential expression for each of the genes derived using EBarrays (Y-axis) to generate a volcano plot to visualize differential expression. Significant and insignificant genes are represented by black and grey diamonds, respectively. Red diamonds represent orthologs of genes identified as constituting the core anaerobic transcriptome of three enterobacterial members grown in the presence of glucose. A set of 20 genes were identified as likely to constitute the minimal core anaerobic transcriptome of the Enterobacteriaceae in the presence of glucose as the carbon source [22]. These 20 genes shared a 1-1-1 orthologous relationship between three members of the Enterobacteriaceae, namely *E*. *coli* K-12-MG1655, *Dickeya* *dadantii* 3937 and *Pectobacterium* *atrosepticum* SCRI1043 and for all of the 20 genes the pattern of expression was similar, the magnitude of change was greater than 3-fold and the genetic architecture was highly conserved. While the exact functions of most of these genes are established in the model organism *E*. *coli* that of few others still remain elusive. Of these 20 genes, 18 were differentially expressed and showed similar pattern of expression in *Y*. *intermedia* in this study. These are *frdABCD* (fumarate reductase), *focA, yfiD*, (pyruvate formate lyase), *adhE* (aldehyde dehydrogenases), *ynfK* (dethiobiotin synthetase)*, hypC* (hydrogenase components), *nrdD* (anaerobic ribonucleotide reductase), *dcuB* (dicarboxylate transporter), *yhbUV* (collagenase-like proteins), *pepT* (peptidase), *ycbJ* (uncharacterized protein), *exbB* (the membrane-spanning protein of the TonB-exbBD complex), *yceJ* (cytochrome), *yceI* (uncharacterized protein). Except for five genes (yfiD, yhbV,ycbJ, yceJ, yceI), all of the remaining 13 genes showed fold changes greater than 3 (our stringent criteria established in a previous study) in *Y*. *intermedia*. The only gene which is present in the core but missing in the differentially expressed set in *Y*. *intermedia* is *nrdG* (anaerobically functioning ribonucleotide reductase).

We obtained RNA from three replicate cultures of *Y. intermedia* grown under either aerobic or anaerobic conditions. After conversion of the RNA to cDNA, each sample was labeled with Cy3 and hybridized to Nimblegen arrays, which were scanned to obtain fluorescence signals for each probe. From this, we derived median signal intensities for each gene, and calculated fold changes between the conditions for each gene as described (methods section). Using an Empirical Bayesian analysis we determined the critical threshold that allowed for a 1% false discovery rate (FDR) and identified 424 genes to be differentially expressed between aerobic and anaerobic conditions (see Table S2 and Figure 1). Of these, 238 were up-regulated and 186 were down-regulated under anaerobic conditions. If we required that genes change expression by at least 3-fold in addition to the statistical significance threshold, 136 genes were identified as differentially expressed (log ratios >1.5 or <-1.5, 66 up-regulated, 70 down-regulated). Except for three non-coding RNAs and one tRNA (t-RNA-Leu) all of the remaining differentially expressed genes were protein-coding.

**Figure 2:**
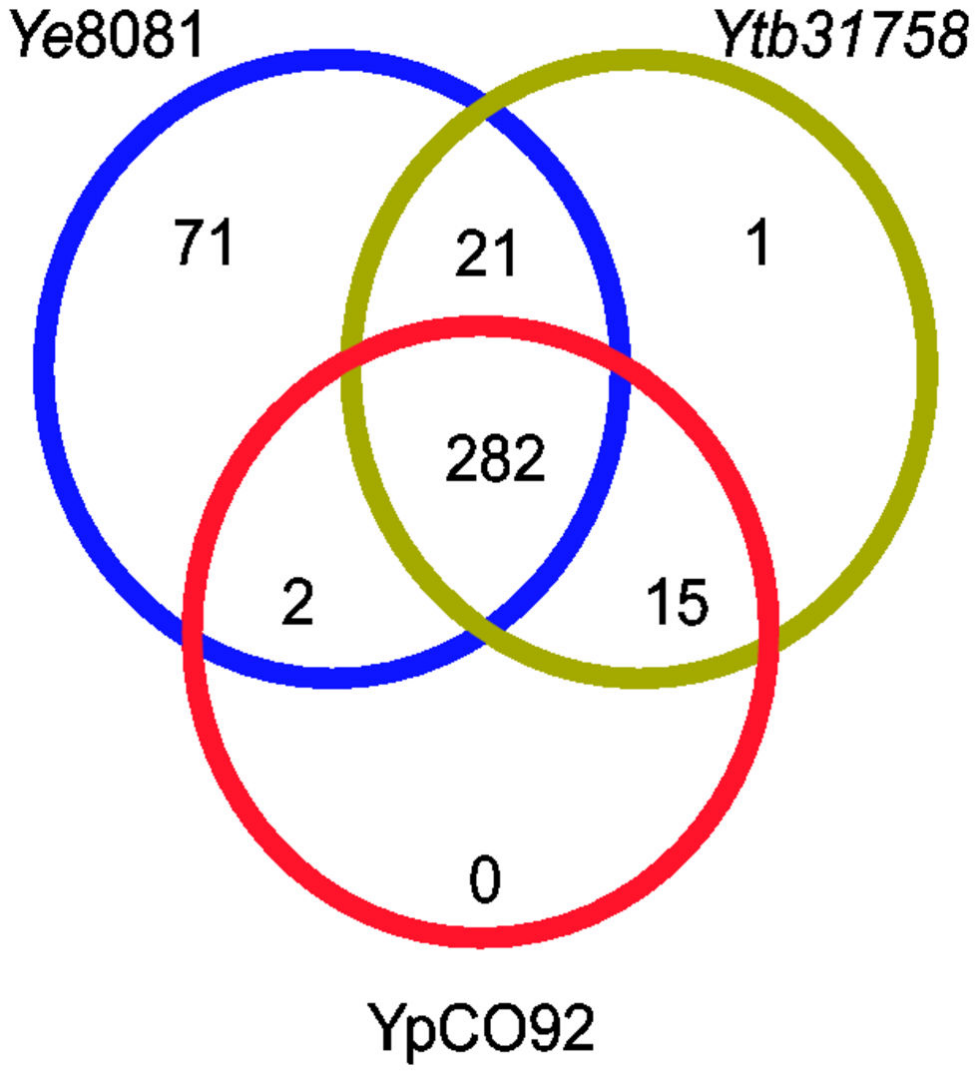
Graphical representation of 392 differentially expressed genes in Y. intermedia which have homologs in at least one of the pathogenic yersiniae. Phylogenprofiler (Integrated Microbial Genomes) was used with analytical settings as described in methods to obtain homologs of *Y*. *intermedia* in other yersiniae. A Venn diagram was built to display the number of differentially expressed genes in *Y*. *intermedia* that have homologs in *Y*. *enterocolitica* (*Ye8081*, blue circle, 376 genes), *Y*. *pseudotuberculosis* (*Ytb31758*, purple circle, 319 genes) and *Y*. *pestis* (*YpCO92*, red circle, 299 genes).

The number of genes differentially expressed in *Y. intermedia* in response to oxygen availability is within the range (5 to 10%) that has been observed for other members of the Enterobacteriaceae under similar experimental conditions [19,22]. The utility of considering the *Y. intermedia* responses to environmental stimuli as indicative of what to expect in the pathogenic Yersinia depends partially on whether differentially expressed genes are conserved among members of the genus. The majority (393 out of 420) of the protein-coding genes that were differentially expressed were shared with at least one of the human pathogenic yersiniae. None of them were shared exclusively with *Y. pestis*. 376 genes were shared with *Y. enterocolitica 8081* and 319 were shared with *Y. pseudotuberculosis* 31758 (Figure 2). The remaining 27 *Y. intermedia* genes were not shared with any of the human-pathogens. Of the 27, only 8 genes were unique to *Y. intermedia* compared to any other yersiniae including other non-pathogenic species (see Table 1). The physiological consequences of gains and losses of genes that respond to changes in oxygen availability in these lineages remain largely unclear at this point.

**Table 1:** Strain specific genes of *Y. intermedia* that are differentially expressed during anaerobiosis and are not present in pathogenic isolates (*Y. enterocolitica* (*Ye8081*), *Y. pseudotuberculosis* (*Ytb31758*) and *Y. pestis* (*YpCO92* genes) of the yersiniae.

**ASAP Feature ID**	**Locus Tag**	**Gene Name**	**Gene Product**	**Fold Change**	**Signal Intensity (Log_2_)**	
					O_2_	w/o_O_2_
AEH-0000354	YintA_01000356		COG3539: P pilus assembly protein, pilin FimA	2.7	12.1	13.5
AEH-0000378	YintA_01000381		Methyl-accepting chemotaxis protein	4.7	10.1	12.3
AEH-0000401	YintA_01000404	*fliC*	flagellar filament structural protein (flagellin)	2.2	14.4	15.5
**AEH-0000514**	**YintA_01000518**		**hypothetical protein**	**4.6**	**10**	**12.2**
**AEH-0001058**	**YintA_01001071**		**Arylsulfatase regulator (Fe-S oxidoreductase)**	**1.7**	**11.4**	**12.1**
**AEH-0001127**	**YintA_01001141**	***yjjW***	**predicted pyruvate formate lyase activating enzyme**	**2.1**	**9.5**	**10.5**
**AEH-0001128**	**YintA_01001142**	***yjjI***	**glycyl radical enzyme**	**4.3**	**10.3**	**12.4**
AEH-0002071	YintA_01002097	*yidP*	predicted DNA-binding transcriptional regulator	1.7	10.2	11
AEH-0002879	YintA_01002903	*cbiN*	synthesis of vitamin B12 adenosyl cobalamide precursor	2.3	11.5	12.7
**AEH-0003024**	**YintA_01003048**		**hypothetical protein**	**3.6**	**10.8**	**12.7**
AEH-0003219	YintA_01003243		hypothetical protein	6.8	10.8	13.5
**AEH-0003291**	**YintA_01003313**	***allA***	**ureidoglycolate hydrolase**	**1.8**	**10.8**	**11.6**
AEH-0003890	YintA_01003888		COG3338: Carbonic anhydrase	2.2	9.9	11.1
AEH-0004003	YintA_01003975		hypothetical protein	1.8	13.3	14.2
AEH-0001136	YintA_01001150		RTX toxins and related Ca2+-binding proteins	-1.9	11	10.1
AEH-0001137	YintA_01001151		RTX toxins and related Ca2+-binding proteins	-3.6	12.9	11.1
AEH-0001138	YintA_01001152		hypothetical protein	-2.6	12	10.6
AEH-0001461	YintA_01001482		putative inner membrane protein	-1.9	12.1	11.1
AEH-0001559	YintA_01001582		putative exported protein	-1.8	11	10.2
AEH-0001560	YintA_01001583		putative ABC transport protein, ATP-binding component	-1.8	12	11.1
AEH-0001869	YintA_01001892	*fhuA*	ferrichrome outer membrane transporter	-10.3	13.7	10.3
AEH-0002349	YintA_01002376		hypothetical protein	-3.1	12.2	10.6
**AEH-0002752**	**YintA_01002777**		**Putative threonine efflux protein**	**-2.3**	**12.2**	**11**
AEH-0002753	YintA_01002778		Siderophore-interacting protein	-3.5	12.1	10.3
**AEH-0003449**	**YintA_01003465**	***mmuM***	**CP4-6 prophage; S-methylmethionine:homocysteine methyltransferase**	**-2.7**	**12.8**	**11.4**
AEH-0003450	YintA_01003466	*mmuP*	CP4-6 prophage; predicted S-methylmethionine transporter	-2	12.3	11.3
AEH-0000386	YintA_01000389	*ybhB*	putative Phospholipid-binding protein	-1.8	12.8	12

Bold fonts represent genes present only in *Y. intermedia* ATCC29909

### Analysis of DE genes

We grouped the 424 differentially expressed genes from *Y. intermedia* into 11 categories according to their predicted cellular functions (Figure 1 and Table S2). The breadth of functional categories covered by this set of genes, ranging from energy metabolism to those associated with specific functions such as chemotaxis and motility, indicates that in *Y. intermedia* O_2_ availability impacts cellular metabolism in a global manner. Below, we describe transcriptional changes for differentially expressed genes associated with these functional categories. In these sections we compare our data to model organism *E. coli* K-12 to determine what knowledge from the well-studied model system can apply to understanding these conditions in *Y. intermedia*. Where relevant, we describe the ortholog differences between *Y. intermedia* and pathogenic yersiniae to identify differences and similarities in their potential response to O_2_.

### 1. Energy Metabolism-Glycolysis, Fermentation and TCA cycle

**Figure 3:**
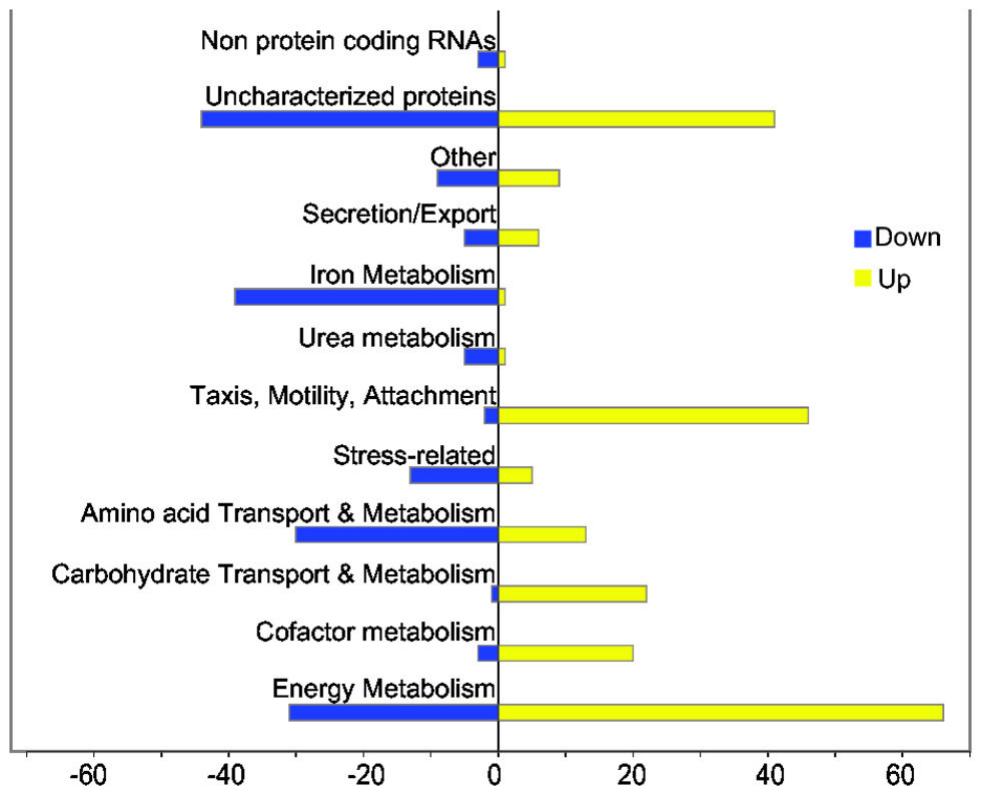
Functional categories of anaerobically up-regulated (yellow) and anaerobically down-regulated (blue) genes. Genes from Table S1 were broadly categorized according to their biological function. Each bar represents the actual number of genes.

Overall, the pattern of expression changes for genes associated with energy metabolism (Table S1) in *Y. intermedia* agrees with observations from studies of other enterobacteria and suggest that *Y. intermedia* performs mixed-acid fermentation under anaerobic conditions with glucose as sole carbon and energy source. This is the preferred mode of energy metabolism in *E. coli* and many other enterobacteria when external alternate electron acceptors are unavailable. Under anaerobic conditions several genes associated with fermentation and anaerobic respiration were up-regulated and those with roles in aerobic metabolism including the TCA cycle were down-regulated (Figure 3 and Table S1). Specifically, genes encoding glycolytic enzymes (*pgi, tpiA, pgk, gpmI, eno, pykA, pykF*) and the phosphoenolpyruvate carboxylase encoding gene (*ppc*) were up-regulated. The fermentative alcohol dehydrogenase *adhE* was also up-regulated. Modulation of glycolytic enzyme gene expression in response to oxygen limitation is indeed well documented in prokaryotic as well as eukaryotic organisms, and the genes and proteins involved are some of the most ancient and highly conserved proteins known [23,24]. The only down-regulated gene associated with glycolysis was *gpmA*. The *E. coli gpmA* ortholog encodes a cofactor dependent phosphoglyceromutase, GpmA. This enzyme has a much lower specific activity than its Non-homologous ISofunctional Enzyme (NISE) GpmI encoded by *gpmI*, which was up-regulated anaerobically [25,26]. Thus, the GpmI isozyme may be preferentially used during anaerobiosis. The organization and up-regulation of genes that participate in the conversion of pyruvate to formate and formate transport under anaerobic conditions (*pflB*, *pflA* and *focA*) resemble that of *E. coli*.

In *Y. intermedia*, the expression pattern of the genes encoding hydrogenases is similar to that of *E. coli*, but the gene organization is not conserved between the two organisms. The *E. coli* genome encodes four distinct hydrogenase gene clusters, named hydrogenase 1, 2, 3 and 4. *Y. intermedia* encodes genes for only two of the hydrogenases which are grouped into two clusters, *hybOABCDEF-hypB-hybG-hypDE* and *hyfABCDEFGHIJK-hydN-fdhF-hyfR*. These correspond to genes necessary for the function and maturation of the *E. coli* Hyd-2 respiratory hydrogenase and the Hyd-4 hydrogenase that is part of the formate hydrogen lyase complex, respectively. All these genes are up-regulated under anaerobiosis both in *Y. intermedia* and in *E. coli*. Hydrogenase activity is necessary for production of energy and growth during the course of infection for a number of animal pathogens and therefore contributes to their virulence [27]. Surprisingly, *Y. pestis* and *Y. pseudotuberculosis* lack any genes encoding hydrogenases [5]. The observation that the genomic architecture of hydrogenases exhibits remarkable heterogeneity among the Enterobacteriaceae [22] and that the expression of hydrogenases is tightly regulated by a number of factors [28] suggest a complex evolutionary history.

The anaerobic stimulon of *Y. intermedia* contains other up-regulated genes with previously demonstrated roles in anaerobic metabolism in *E. coli* and other enterobacteria. These include *dcuA*, *dcuB* (dicarboxylate transporters), *pck* (phosphoenolpyruvate carboxykinase), *ynfK* (predicted dethiobiotin synthetase), *yhbUV* (proteases) and *pepT* (peptidase).

Like *E. coli*, the *Y. intermedia* genome also encodes a repertoire of reductases (*frdABCD*, *dmsABCD and its paralogs*, *torCA*) required for the utilization of alternate electron acceptors such as fumarate, dimethyl sulfoxide (DMSO) and trimethylamine N oxide (TMAO), for respiration under oxygen-limiting conditions. Expression of all of these genes was up-regulated anaerobically in *Y. intermedia*, similar to *E. coli*. In the case of fumarate reductase, genes required for biosynthesis of its menaquinone cofactor, namely, *menF* and *menDHBCE* [29], were also up-regulated. In addition, genes for tetrathionate respiration (*ttr*) are present in *Y. intermedia* but they were not differentially expressed under our experimental conditions. It is possible that *Y. intermedia* up-regulates *ttr* genes in the inflamed human gut where tetrathionate is produced from thiosulfate [30].

Other widely used electron acceptors among the enterobacteria are nitrate and nitrite, which are abundant in environments that *Y. intermedia* inhabits. The *Y. intermedia* genome encodes a membrane bound nitrate reductase (*narGHJI*) and a periplasmic nitrate reductase (*napFDABC*). Consistent with the absence of nitrate in our experimental media, these genes remained unaffected under anaerobic conditions and are presumed to require the activation of the nitrate dependent two-component sensor kinase /response regulator NarL/P or NarX, (NarQ is not present in *Y. intermedia*). This is consistent with what was observed in *E. coli* K-12 that harbors genes for three different nitrate reductase systems, none of which were up-regulated under anaerobic conditions similar to those used here [19].

Genes encoding proteins involved in or related to the TCA cycle (*aceAB, aceEF, acnB*, *fumC*, *icd, mdh*, *sdhABCD, sucA, lpd, lipAB, pdhR*) and genes encoding components of the aerobically active cytochrome oxidase complex (*cyoABCDE and yceJ*) were all down-regulated more than 3- fold as expected for genes encoding enzymes associated with aerobic metabolism.

In *E. coli*, the best-studied member of the Enterobacteriaceae, FNR, ArcA/B, NarL/P and NarX/Q are the major global regulatory systems which govern anaerobic expression of genes [31]. Except for NarQ, orthologs for all these regulators are present in *Y. intermedia*. None of these regulators showed differential expression under our experimental conditions, similar to what was observed for these genes in *E. coli*. This is perhaps not surprising since the activity of the regulators is largely determined by post-transcriptional events.

### 2. Carbohydrate uptake and metabolism

The expression pattern of genes associated with carbohydrate uptake suggests that *Y. intermedia* can utilize diverse carbohydrates under anaerobic conditions. Specifically, genes for the uptake of glucose (*ptsG, glk*), fructose (*fruBKA*), fructose/ mannose/N-acetyl galactosamine (*manX, manZ*) and sorbitol/glucitol (*srlAEB*) were all up-regulated in *Y. intermedia*. Except for *glk*, these genes encode phosphoenolpyruvate dependent phosphotransferase systems (PTS), which couple transport of sugars and sugar alcohols to their phosphorylation for further metabolism. Considering that only glucose was present in the medium we used, the up-regulation may be indicative of cells preparing themselves to utilize a wide range of carbon sources when they become available [32]. Transcripts of *ptsG*, *glk*, *fruBKA* in *E. coli* showed statistically significant yet less than 1.5- fold up-regulation during anaerobiosis. It is possible that the PTS systems of *Y. intermedia* couple carbon source sensing to anaerobic global regulatory mechanisms [33].

### 3. Metabolism and transport of amino acids and peptides

Most of the genes associated with biosynthesis (*metA*, and *metBL, metF*), transport (*metN, metI, mmuM, mmuP*), and regulation (*metR*) of the sulfur-containing amino acid methionine were strongly down-regulated under anaerobic conditions. Either of two isozymes of methionine synthase, namely, MetE and MetH, can catalyze the last step of methionine biosynthesis, which is the conversion of homocysteine to methionine. MetE is cobalamin-independent and MetH is cobalamin-dependent. Both genes were down-regulated under anaerobic conditions. The *Y. intermedia* genome contains the entire gene repertoire required for cobalamin biosynthesis which was curiously up-regulated under anaerobic conditions in our experiments (discussed under cofactor metabolism below). These genes were all unaffected by oxygen availability under similar conditions in *E. coli* which is missing most of the genes for cobalamin biosynthesis.

Some enterobacteria encode genes for the methionine salvage pathway that recycles methylthioadenosine, a product of polyamine [34] and N-acylhomoserine lactone biosynthesis [35]. back to methionine. Genes encoding components involved in this pathway (*mtnK, mtnABCDE, yafV*) were down-regulated in *Y. intermedia* under anaerobic conditions. This pathway does not appear to be complete in most strains of *E. coli* [36].

Cysteine biosynthesis genes were up regulated (*cysK, cysH*). Lysine biosynthesis genes (*dapB, lysA*) were down-regulated. Genes associated with degradation of serine/threonine (*ydfG*) were up regulated while degradation of D- and L- alanine (*dadAX*) was down regulated. Transcripts for most of these genes remained largely unaffected in *E. coli*.

Genes involved in the transport of peptides (*dppABCDF, tppB*) and two serine-transporter genes (*sdaC and sst*) and were up-regulated anaerobically in *Y. intermedia*. *E. coli* also up-regulates transcripts of these genes during anaerobiosis. These observations suggest that uptake of some peptides and certain amino acids is a conserved response in the enterobacteria while uptake of others are lineage-specific. Roles of these molecules in supporting various cellular processes during oxygen limitation are beginning to be understood. For example, it has been shown that *E. coli* could degrade serine to remain motile under anaerobiosis [37]. A strain of the uropathogenic *P. mirabilis* lacking DppA was attenuated *in vivo* [38]. In *E. coli* and *Salmonella Typhimurium* up-regulation of dpp peptide transporters observed under anaerobic conditions is FNR-mediated [19,20]. It remains to be seen how these processes are regulated in the Yersinia.

### 4. Co-factor Metabolism

Cobalamin (coenzyme B12) is a chemically complex cofactor that is unevenly distributed among modern life-forms [39]. It can be synthesized via aerobic and anaerobic pathways that mainly differ in the step of cobalt insertion [40]. In our experiments, anaerobic up-regulation of *cbiACDEFGHJKLMNPQST, cobD, pocR* encoding the enzymes that participate in the anaerobic biosynthetic pathway was observed. *Y. intermedia* genes for coenzyme B12 (cofactor cobalamin) biosynthesis are orthologous to those of *Y. enterocolitica* and *Salmonella*. In these and other enterobacteria, the primary function of B12-dependent reactions is to support anaerobic metabolism of small molecules such as ethanolamine, propanediol and glycerol [39]. In our experiments, expression of genes for none of these other B12-dependent processes was affected by O_2_ availability, probably because activation of these genes requires the presence of the respective small molecules [39]. The B12-dependent genes appeared to be moderately expressed (with average log_2_ expression values near10 units) regardless of availability of oxygen.

### 5. Stress-Related Functions

Several genes encoding proteins that mediate tolerance/resistance to specific stressors such as oxidative stress, carbon-starvation, acid stress and osmotic stress as well as those to general stress conditions were oxygen-responsive in *Y. intermedia*. Except for *katA*, *sodB, uspA* and one of the carbonic anhydrases (YintA_01003888), all others were down-regulated under anaerobic conditions, indicating that these genes are primarily involved in stress-related functions associated with aerobic cellular metabolism. The possible biological significance of changes for some of these genes is discussed below.

#### Oxidative stress

The functionally equivalent genes, *sodB and sodA*, which encode cytoplasmic superoxide dismutases, show divergent expression patterns. Similar to that observed in *E. coli*, transcripts of *sodB* are up-regulated and that of *sodA* are down-regulated under anaerobic conditions. The up-regulation during O_2_ limitation of sodB that encodes the iron-requiring SodB enzyme in *Y. intermedia* is is similar to the response seen for the gene in the three human-pathogenic yersiniae [17]. Bacterial mutants compromised for the activity of SodB are almost always attenuated in virulence, which suggests a universal role for SodB in the pathogenicity of these organisms. The similar trends in expression in non-pathogenic bacteria (*E. coli and Y. intermedia*) suggest that SodB might contribute to general survival of bacteria under anaerobiosis. In contrast, the manganese cofactored SodA is required for full virulence of *Y. enterocolitica* [41] but not *Y. pseudotuberculosis* or *Y. pestis*. It seems therefore that requirement of SodA for virulence of pathogenic *yersiniae* may be species-specific and related to differences in their infection process. Differences in the oxidative stress environment encountered by the pathogens in their hosts might account for the variability in phenotypic effects of *sodA* mutations.

#### Acid Stress

The down-regulation of transcripts of genes involved in glycine decarboxylation (*gcvTHP*) and glutamate decarboxylation (orthologs of *gadA*/*gadB, gadC* and *glsA1/ybsA*), both of which are associated with acid-stress tolerance mechanisms in other bacteria [42] was somewhat surprising. In *E. coli gadABC* are strongly upregulated under anaerobic conditions [43,44,19], apparently to remove intracellular protons that are generated from the weak acids that accumulate from fermentative metabolism. On the other hand, in two enterobacterial phytopathogens, namely, *Pectobacterium atrosepticum* SCRI1043 and *Dickeya dadantii* 3937, genes encoding glutamate decarboxylase are entirely missing. The production of butanediol as suggested by the up-regulation of its genes (*budABC*) during anaerobiosis in these phytopathogens [22] may compensate for this loss by minimizing the production of acidic end products. Even though *budABC* orthologs are present in *Y. intermedia*, their expression remained unaffected in our experiments. In this context, it is noteworthy that levels of *aspA* were elevated under anaerobic conditions in our experiments similar to the trend in *E. coli*. Recently, Hu et al [45] reported a novel aspartate dependent acid tolerance mechanism in *Yersinia* in which the ammonia released by deamination of aspartate by AspA elevates cytoplasmic pH. The transcription pattern of urease, which is known to be involved in acid stress response in *Y. enterocolitica* is discussed later. One gene for carbonic anhydrase (YintA_01003888) showed higher transcription under anerobic conditions in *Y. intermedia*. Carbonic anhydrases catalyze the reversible hydration of CO2 and may serve to regulate pH homeostasis under anaerobic conditions. They are important for virulence for several pathogenic bacteria and are targets for therapeutic agents [42]. Taken together these observations suggest substantial diversity in acid tolerance mechanisms and their regulation in the enterobacteria, but provide little understanding of their precise biochemical roles in growth under different conditions.

#### General Stress

Universal stress proteins (USPs) are induced by a variety of stressors and are essential for anaerobic survival in several bacteria [46]. In addition, they are thought to have niche-specific roles in different enterobacteria including the yersiniae. For example, UspA of *Y. enterocolitica* was proposed to be important within insect hosts based on a comparative study between this genome and that of an insect endosymbiont, *Photorhabdus luminescens* [47]. The *uspA* gene was up-regulated in *Y. intermedia* but the adjacent, divergently transcribed, *uspB* gene was down-regulated. In *E. coli* the *uspB* gene is regulated by RpoS whereas *uspA* is not. It is unknown what factors regulate these genes in the *Yersinia*, but the basic gene organization is conserved.

There are several other differentially expressed genes that may be responsible for larger-scale modulations of cellular processes in response to stressful environments. These include the gene encoding the alternative sigma factor RpoS and *rsd*, a regulator of sigma D, both of which were down-regulated under anaerobic conditions. RpoS is proposed as the master-regulator in deciding the trade-off between stress-survival and nutritional competence or the SPANC balance (self-protection and nutritional competence) [48]. It directly or indirectly modulates expression of a large number of genes involved in stress-resistance, maintenance metabolism, programmed cell death and virulence under a variety of suboptimal environments [49]. Because of the pleiotropic effects that RpoS may have on cellular processes, it is likely that the down-regulation of its gene during anaerobiosis in our experiments is due to integration of multiple regulatory signals. It is thought that the RpoS regulon may be different in even closely related enterobacteria [48]. With respect to virulence, the requirement of RpoS among enterobacteria is varied. It is required for virulence in *Salmonella* [50], *Citrobacter rodentium* [51] and in *E. coli* O157:H7 [52], but not in *Y. enterocolitica* [53].

Another stress-related down-regulated gene is *cpxP* whose product regulates proteolysis in energy-poor environments [54]. CpxP is an important component of the envelope-stress response, some components of which are controlled by RpoS.

### 6. Taxis, Motility and Attachment

*Y. intermedia* is motile and its genome encodes more than 20 genes that may have roles in chemotaxis according to their annotated functions and more than 40 genes are involved in the assembly and synthesis of flagella. Sixteen (12 MCPs (Table S1, Taxis) and 4 putative MCPs (Table S1, uncharacterized proteins)) out of the 20 chemotaxis-associated genes were up-regulated under anaerobic conditions suggesting that tactic responses may be important during anaerobic growth or survival of *Y. intermedia*. Among these, several have established roles in chemotaxis (*cheZY*, *cheBR*, an ortholog of *tap* (YintA_01000447), *cheA*, *cheW* and *tar*), and, are located within the region containing the flagellar genes. The others include an ortholog of *aer*, the aerotaxis receptor encoding gene that is involved in energy taxis in many Gram-negative bacteria, *tsr* which encodes a serine chemoreceptor in *E. coli* and two other genes which probably function in tactic responses, namely an ortholog of *trg*, and a gene which encodes a protein similar to CheV [55]. Almost all of the flagellar genes, including the regulatory genes *flhDC* and the flagellar motor genes *motAB*, were up-regulated with an average fold change of ~2 under anaerobic conditions.

Three adhesins are shared by the enteropathogenic yersiniae, namely, invasin, YadA and Ail that enable them to adhere to host-cells. *Yersinia pestis* lacks invasin and *YadA* but encodes autotransporter adhesins, pH6 antigen and plasminogen activator that mediate adhesion [56]. None of these are present in *Y. intermedia*. However, two genes that were down-regulated in our experiments may have a role in attachment. One of the two encodes a protein that is orthologous to the *Y. enterocolitica* pili protein MrpA (mannose-resistant/*Proteus* like) [57]. MrpA belongs to the π-clade of pili proteins according to a recent classification system based on phylogenic analyses, and is present in beta- and gamma-proteobacteria [58]. MrpA mediates mannose- sensitive haemagglutinin activity in uropathogenic *Proteus mirabilis* [59] and its expression is affected by temperature and aeration [60]. The other down-regulated gene, *crl*, encodes a transcriptional regulator. In the avian pathogenic *E. coli chi* 7122, *crl* plays a non-essential role in the production of curli, hemagglutination and fibronectin-binding during anerobiosis [61].

The up-regulation of several taxis and motility-related genes and down-regulation of genes required for attachment suggests that in *Y. intermedia*, anaerobiosis may favor a planktonic life-style. These expression patterns are similar to those of *E. coli* suggesting that activation of motility functions may be an important general response to anaerobiosis.

### 7. Iron Metabolism

In *Y. intermedia*, more than thirty genes associated with iron uptake and storage were down-regulated under anaerobic conditions. These genes are involved in synthesis and uptake of aerobactin [62,63] or a related siderophore (*iucABCDiutA* [64],, transport of enterobactin (*fepA*), transport of ferric iron (*fhuC*, *Y. pestis fitABCD* orthologs, *yfuAB, yfeABCD, exbBD, tonB*), uptake of ferrous iron (*efeBOU*), and storage of iron (*ftn*, *bfd*). Genes involved in the transport of hemin were also down-regulated (*hemP, hmuRSTUV*). The sole up-regulated gene in this category *yfeX* was proposed to encode an enzyme that is able to dechelate iron from heme [65], but actually appears to have been misclassified and is now thought to be an anaerobic porphyrinogen oxidase [66].

### 8. Urea Metabolism

Under the anaerobic conditions used in our experiments, the structural genes for urease (*ureABC*) were down-regulated more than 2-fold while the accessory genes (*ureEF*) that are necessary for the incorporation of nickel in the enzyme were less than 2-fold down-regulated. Urease activity is considered to be important for the saprophytic life-style of yersiniae since it hydrolyzes urea present in soil and water environments, releasing NH3 for a nitrogen source. It is likely that urease activity is crucial for *Y. intermedia* under specific circumstances, for example during growth in human urine.

Urease also contributes to virulence in several bacteria [67]. However, unlike ureases from other enterobacteria, the enzyme from *Y. enterocolitica* was not induced by urea or nitrogen limitation. It is functional at very low concentrations of urea and showed optimal activity at low pH (3.5 to 4.5), suggesting that *Y. enterocolitica* expresses its urease during acid stress and low-urea conditions such as that present in the stomach of mammalian hosts [68]. If the *Y. intermedia* urease functions during acid stress, then its down-regulation follows the trend of other acid-stress related genes.

### 9. Other

Three up-regulated genes in *Y. intermedia* encoded proteins similar to those encoded by the *Salmonella srfABC* operon. These genes are found in most *Salmonella* and are important for virulence, although their exact functions remain to be elucidated. Similar proteins are found in most Yersinia genomes and the genomes of phytopathogenic enterobacteria, but not in genomes of most other sequenced enterobacteria. The *srfC* gene is similar to an ADP ribosyltransferase that is secreted by the Hrp type III secretion system of *Pseudomonas syringae* pv. *tomato* DC3000 [69]. We did not detect differential expression of type III secretion system genes in *Y. intermedia*. In *S. Typhimurium*, *srfABC* is regulated by the two-component system SsrAB as well as by FNR and was up-regulated anaerobically [70,20]. Expression of this operon was also shown to be FlhDC regulated [71] suggesting a link to flagellar biosynthesis, although a mutant showed normal motility.

A cluster of four toxin-related genes were down-regulated under anaerobic conditions in *Y. intermedia*. These genes are homologous to those encoding a serralysin-like metalloprotease, a protease inhibitor and secretion system that are involved in pathogenesis in the fish pathogen *Y. ruckeri* that causes the enteric red mouth disease of salmonids, where they were expressed in a temperature dependent manner [72]. They are also regulated by osmolarity and pH but regulation due to oxygen availability has not been previously reported. Interestingly one of the four genes in the *Y. intermedia* locus encodes an additional toxin-like protein that is not present in the *Y. ruckeri* genome.

Genes involved in fatty acid metabolism (*fadB, fadL*) were up-regulated. In *E. coli* a set of paralogs to the *fad* genes support anaerobic growth on fatty acids [73]. These genes are present in *Y. intermedia* as well, but are not differentially expressed.

The *efp* gene encoding elongation factor P was upregulated. and the protein folding related gene *fklB* was also up-regulated, The *Y. pseudotuberculosis fklB* gene was shown to have peptidylprolyl cis-trans isomerase activity and a mutant was attenuated for virulence in mice [74].

Genes that are probably involved with copper efflux *cusA*, *cusB*, *cusF* and *copA* were significantly up-regulated as well as the first gene in the *cus* operon that encodes the outer membrane component of the transporter, but differs significantly in sequence from the *E. coli cusC* sequence. In *E. coli* the *cusCFBA* genes were shown to be part of a group of genes repressed by FNR, but not oxygen-regulated under standard anaerobic growth conditions [19].

DNA-damage-repair related genes (*recN*, *dinB, dinI, umuD*) and nucleotide metabolism genes (*udp*, *ycgR*) were down-regulated. Genes for metabolism of uracil (*uraA, upp*) were up-regulated.

### 10. Uncharacterized and Hypothetical proteins

A significant proportion of the differentially expressed genes encoded proteins with vaguely predicted functions or with no predicted function indicating that many aspects of the response to oxygen availability in *Y. intermedia* remain to be understood. The differentially expressed genes with functional annotations included those involved in energy metabolism (oxidoreductases), transport or signal perception (membrane-associated proteins, MCPs), regulation (DNA-binding proteins) and iron uptake (siderophore-related).

### 11. Non-coding RNAs

Post-transcriptional regulation mediated by small regulatory RNAs may be critical during anaerobiosis in *Y. intermedia* as suggested by strong differential expression of three small RNA-encoding genes in our experiments. The small regulatory RNA Fnr*S* that has been previously demonstrated to be activated under anaerobiosis was highly up-regulated in our experiments. FnrS is postulated to contract the FNR regulon and its primary targets are genes that are not required during anaerobic metabolism [75,76]. Orthologs in *E. coli* for some of the differentially expressed genes in *Y. intermedia* are members of the FnrS regulon (*yceI, gpmA, folE, metE, sodB*). Two other small RNAs, namely RyhB and RyhB2 were highly down-regulated. These sRNAs which are expressed under iron-limiting conditions are regulated by Fur in *E. coli* [77]. In *E. coli* RyhB increases the available free iron pool to enzymes that are critical for cellular metabolism by down-regulating some of the non-essential iron-requiring enzymes [78].

## Summary and Conclusions

Our transcriptome analysis offers insights into how *Y. intermedia* regulates expression of its versatile biochemical repertoire in response to oxygen availability. The transcription patterns of a majority of genes in *Y. intermedia* were similar to that of homologs from the model organism *E. coli*. However, notable differences were found with respect to genes involved in amino acid metabolism, stress tolerance, motility response and iron metabolism. In our previous study [22] comparing 3 genera of enterobacteria under the same growth conditions used here, we found only 20 genes with orthologs in all three genomes that were significantly differentially expressed in all 3 organisms. In this study 18 of those 20 genes showed similar expression differences (only 13 meet our most stringent criteria for differential expression). The small number of genes in this core of the anaerobic stimulon suggests that the key changes required to grow anaerobically are accomplished by a small number of factors, or more likely, that the biochemistry of anaerobic growth in each species in governed by a combination of conserved ancestral genes that show conserved regulation, conserved genes that are differentially regulated between species, and genes that are variably present/absent from taxa due to horizontal acquisition and deletion.

*Y. intermedia*, like *E. coli*, expresses genes for utilizing alternative electron acceptors, alternative carbon sources and motility and chemotaxis, even under the simple anaerobic growth conditions used in our experiment that contains only glucose as a carbon source and no alternate electron acceptors. These findings may not be completely reflective of certain anaerobic conditions of other *Yersinia* spp. For example, the flea midgut environment is not abundant in glucose [79]. Furthermore, *Y. pestis* carbohydrate metabolism is not expressed during such conditions [80]. *Y. pestis* predominately relies upon oligopeptides as the carbon/energy source during growth in the flea. Additional regulatory factors such as cAMP receptor protein (CRP) may be active during the utilization of alternate carbon sources which may influence anaerobic energy metabolism [81]. Perhaps repression of expression of these genes occurs under other growth conditions, Temperature is a significant factor governing gene expression in the yersiniae. This analysis strictly assessed anaerobic growth conditions at 28°C which does not reflect oxygen-limitation during human infection as associated with macrophage intraphagosomal survival or infection of the human ileum. The anaerobic transcriptome may be significantly altered at 37°C. Additional experiments under anaerobic conditions that include alternative electron acceptors and/or carbon sources in the growth medium and carried out at 37°C would be useful for dissecting the regulatory pathways involved in gene expression in the yersiniae.

The *Yersinia* are of particular biomedical interest because of the human pathogens in the genus that include that agent of the plague, *Y. pestis*. For the purposes of studying molecular pathogenesis of disease, development of surveillance and detection methods and understanding the evolution of virulence it is extremely important to study genome and transcriptome differences among *Yersinia* species. The utility of species such as *Y. intermedia* as surrogates for more dangerous pathogens in experiments or as tools for exclusion of samples as dangers likewise relies on an evaluation of the degree of conservation of gene and expression differences among species.

## Methods

### Bacterial Growth and RNA Extraction

We grew *Yersinia intermedia* strain ATCC29909 (Figure S1) in MOPS minimal medium supplemented with 0.1% glucose at 28°C. MOPS buffer (M2101) and dipotassium phosphate (M2102) were purchased from Teknova, Inc., USA. Overnight cultures of the bacterium were diluted to an O.D.600 of 0.05 in fresh medium and cultured in a gas sparging system to permit precise control over the mixtures of O2, N2 and CO2. Cultures were grown to early log phase under aerobic (70% N2, 25% O2 and 5% CO2) and anaerobic (95% N2 and 5% CO2) conditions. 20 ml samples (Aerobic cultures O.D.600 =0.45, Anaerobic cultures O.D.600 =0.25,) were collected in tubes containing 2 ml phenol-ethanol. RNA was extracted using the hot-phenol method as previously described [82]. Quality of RNA samples was assessed using the Agilent Bioanalyzer 2100 nanochip system (Agilent Technologies).

### Microarray Design and Hybridization

Sequences and annotations for predicted protein-coding genes for Y. intermedia ATCC29909 were obtained from the ASAP database [83]. Oligonucleotide arrays were synthesized by Nimblegen Inc. for which 320358 genome-specific probes were selected using ChipD using the following settings, target melting temperature: 78 °C, target probe length: 40 to 70 -mers, interval size: 12 [84]. The custom-designed whole-genome-tiled arrays contained ~320,000 probes representing 3953 genes that included 3887 protein coding genes (and 18 likely pseudogenes), 12 non-coding RNAs and 36 tRNAs. Data from probes corresponding to intergenic regions (and some pseudogenes, rRNA genes and unannotated genes) of the genome were not considered in the present analysis.

Procedures described in the Nimblegen Arrays User’s guide were followed for cDNA synthesis, labeling and hybridization (http://www.nimblegen.com/products/lit/expression_userguide_v5p0.pdf). Arrays were scanned on an Axon scanner at 532 nm and signals were extracted using NimbleScan software (NimbleGen Inc. USA). Our arrays did not have probes for regions of sequence that total to ~100 kbp which is present in the recently updated version of the sequence at Genbank (NZ_AALF00000000).

### Analysis of Gene Expression Data

Signals from the 3 replicates (Table S2) of the hybridization experiments from each organism were normalized using RMA [85] implemented in the NimbleScan software and imported into a custom MicroSoft Access database. The median of signals from multiple probes (from coding and non-coding strand) for each individual gene was calculated using R, from which log_2_ expression values were derived. To determine directional changes in gene expression, log_2_ ratios were determined by calculating the difference between the log_2_ median anaerobic signal and log_2_ median aerobic signal. To identify differentially expressed genes between aerobic and anaerobic growth, an empirical Bayesian analysis, EBArrays [86] was executed within the free statistical analysis software package R [87] and Bioconductor v2.1 [88]. Aerobic and anaerobic growth conditions from each dataset were used to define two patterns: equivalent (111,111) and differential (111,222) expression. The posterior probability for each pattern was calculated using a hierarchical log-normal normal expression model with the conditional false discovery rate (cFDR) at 0.01 to determine the appropriate threshold (cFD(τ)). The critical threshold for the data set was 0.8994049. The raw data and processed data for the experiments are deposited in the National Center for Biotechnology Information Gene Expression Omnibus database (http://www.ncbi.nlm.nih.gov/geo/query/acc.cgi?acc=GSE50043).

### Comparative Analysis

Presence or absence of homologs was determined using automated options available at the Integrated Microbial Genomes database with the following settings: Max. E value: 1e-5, Algorithm: Present/Absent Homologs, Minimum percent identity: 60, Exclude pseudogenes: Yes (http://img.jgi.doe.gov/cgi-bin/w/main.cgi?section=PhylogenProfiler&page=phyloProfileForm).

## Supporting Information

Figure S1 **Growth of *Y. Intermedia* in minimal medium supplemented with 0.1% glucose under aerobic conditions (Above) anaerobic conditions (Below).** Arrows indicate time of sampling.
(EPS)

Table S1 **Differentially expressed genes in specific functional categories *Y. Intermedia* in minimal medium supplemented with 0.1% glucose under aerobic conditions and anaerobic conditions.**
(DOC)

Table S2 **Expression pattern of genes in *Y. Intermedia* in minimal medium supplemented with 0.1% glucose under aerobic conditions and anaerobic conditions.**
(DOC)
