# Supplementary material for: Transcriptome Changes Associated with Anaerobic Growth in Yersinia intermedia (ATCC29909)
Source: PLoS One. 2013 Oct 7;8(10):e76567. doi: 10.1371/journal.pone.0076567 (PMC3792023; doi:10.1371/journal.pone.0076567)
Supplement: Table S1 — Differentially expressed genes in specific functional categories Y. Intermedia in minimal medium supplemented with 0.1% glucose under aerobic conditions and anaerobic conditions. (DOC) [file pone.0076567.s002.doc]

| Functional Category/Gene Product | *Gene Name* | Fold Change | O_2_ | w/o_O_2_ | ASAP Feature ID | Locus Tag |
| --- | --- | --- | --- | --- | --- | --- |
| **1. Energy Metabolism** |  |  |  |  |  |  |
| *Anaerobic Respiration* |  |  |  |  |  |  |
| oxidoreductase subunit | *dmsA* | 8.1 | 10.1 | 13.2 | AEH-0000582 | YintA_01000586 |
| oxidoreductase, Fe-S subunit | *dmsB* | 5.9 | 10.2 | 12.7 | AEH-0000583 | YintA_01000587 |
| dimethyl sulfoxide reductase, anaerobic, subunit C | *dmsC* | 4.3 | 10.9 | 13.1 | AEH-0000584 | YintA_01000588 |
| twin-argninine leader-binding protein for DmsA and TorA | *dmsD* | 2.0 | 11.2 | 12.1 | AEH-0000585 | YintA_01000589 |
| dimethyl sulfoxide reductase, anaerobic, subunit A | *dmsA* | 3.3 | 10.3 | 12.0 | AEH-0001090 | YintA_01001104 |
| dimethyl sulfoxide reductase, anaerobic, subunit B | *dmsB* | 2.4 | 10.5 | 11.7 | AEH-0001091 | YintA_01001105 |
| putative dimethyl sulfoxide reductase chain C protein | *dmsC* | 1.9 | 9.5 | 10.4 | AEH-0001092 | YintA_01001106 |
| cytochrome C-type protein | *torC* | 2.5 | 10.1 | 11.4 | AEH-0003734 | YintA_01003740 |
| putative molybdopterin-containing oxidoreductase | *torA* | 1.9 | 10.1 | 11.0 | AEH-0003735 | YintA_01003741 |
| putative pyruvate-flavodoxin oxidoreductase | *nifJ* | 1.7 | 11.0 | 11.7 | AEH-0000693 | YintA_01000699 |
| *Fermentation* |  |  |  |  |  |  |
| pyruvate formate lyase I, induced anaerobically | *pflB* | 7.9 | 12.3 | 15.3 | AEH-0000977 | YintA_01000990 |
| pyruvate formate lyase activating enzyme 1 | *pflA* | 2.7 | 10.2 | 11.7 | AEH-0000978 | YintA_01000991 |
| pyruvate formate lyase subunit | *yfiD* | 2.4 | 10.7 | 12.0 | AEH-0001743 | YintA_01001766 |
| predicted pyruvate formate lyase activating enzyme | *yjjW* | 2.1 | 9.5 | 10.5 | AEH-0001127^s^ | YintA_01001141 |
| glycyl radical enzyme | *yjjI* | 4.3 | 10.3 | 12.4 | AEH-0001128^s^ | YintA_01001142 |
| hypothetical protein |  | 3.6 | 10.8 | 12.7 | AEH-0003024^s^ | YintA_01003048 |
| alcohol dehydrogenase class III/glutathione-dependent formaldehyde dehydrogenase | *frmA* | 1.9 | 12.3 | 13.2 | AEH-0001315 | YintA_01001331 |
| fused acetaldehyde-CoA dehydrogenase and iron-dependent alcohol dehydrogenase and pyruvate-formate lyase deactivase | *adhE* | 4.2 | 13.5 | 15.6 | AEH-0002389 | YintA_01002416 |
| fumarate reductase (anaerobic), membrane anchor subunit | *frdD* | 3.3 | 11.7 | 13.5 | AEH-0003040 | YintA_01003065 |
| fumarate reductase (anaerobic), membrane anchor subunit | *frdC* | 6.1 | 10.9 | 13.6 | AEH-0003041 | YintA_01003066 |
| fumarate reductase (anaerobic), Fe-S subunit | *frdB* | 7.3 | 10.5 | 13.4 | AEH-0003042 | YintA_01003067 |
| fumarate reductase (anaerobic) catalytic and NAD/flavoprotein subunit | *frdA* | 8.3 | 10.9 | 13.9 | AEH-0003043 | YintA_01003068 |
| fumarate hydratase class I, anaerobic | *fumB* | 2.3 | 11.3 | 12.6 | AEH-0000565 | YintA_01000569 |
| formate transporter | *focA* | 7.2 | 11.7 | 14.6 | AEH-0000976 | YintA_01000989 |
| C4-dicarboxylate antiporter | *dcuA* | 1.8 | 10.5 | 11.3 | AEH-0003030 | YintA_01003054 |
| C4-dicarboxylate antiporter | *dcuB* | 6.2 | 9.9 | 12.6 | AEH-0003728 | YintA_01003735 |
| Uroporphyrinogen-III methylase | *cysG* | 2.1 | 10.3 | 11.3 | AEH-0001918 | YintA_01001942 |
| nitrite transporter | *nirC* | 1.7 | 10.6 | 11.4 | AEH-0001919 | YintA_01001943 |
| phosphoenolpyruvate carboxykinase | *pck* | 2.4 | 11.3 | 12.5 | AEH-0003763 | YintA_01003768 |
| predicted dethiobiotin synthetase | *ynfK* | 3.6 | 10.1 | 12.0 | AEH-0000616 | YintA_01000620 |
| *Hydrogenases* |  |  |  |  |  |  |
| carbamoyl phosphate phosphatase, hydrogenase 3 maturation protein | *hypE* | 2.0 | 9.7 | 10.7 | AEH-0001848 | YintA_01001871 |
| protein required for maturation of hydrogenases | *hypD* | 2.4 | 9.9 | 11.2 | AEH-0001849 | YintA_01001872 |
| hydrogenase 2 accessory protein | *hybG* | 2.8 | 10.5 | 11.9 | AEH-0001850 | YintA_01001873 |
| GTP hydrolase involved in nickel liganding into hydrogenases | *hypB* | 3.0 | 9.7 | 11.3 | AEH-0001851 | YintA_01001874 |
| protein involved with the maturation of hydrogenases 1 and 2 | *hybF* | 3.0 | 9.5 | 11.1 | AEH-0001852 | YintA_01001875 |
| hydrogenase 2-specific chaperone | *hybE* | 2.8 | 9.0 | 10.5 | AEH-0001853 | YintA_01001876 |
| predicted maturation element for hydrogenase 2 | *hybD* | 2.4 | 9.4 | 10.7 | AEH-0001854 | YintA_01001877 |
| Fe-S-cluster-containing hydrogenase components 2 | *hydN* | 3.4 | 10.4 | 12.2 | AEH-0003025 | YintA_01003049 |
| formate dehydrogenase-H, selenopolypeptide subunit | *fdhF_p* | 3.8 | 10.5 | 12.5 | AEH-0003026 | YintA_01003050 |
| formate dehydrogenase-H, selenopolypeptide subunit | *fdhF_p* | 2.4 | 10.4 | 11.6 | AEH-0003027 | YintA_01003051 |
| formate dehydrogenase-H, selenopolypeptide subunit | *fdhF* | 2.9 | 10.4 | 11.9 | AEH-0001326 | YintA_01001343 |
| formate dehydrogenase-H, [4Fe-4S] ferredoxin subunit | *hydN* | 2.7 | 9.2 | 10.6 | AEH-0001327 | YintA_01001344 |
| predicted processing element hydrogenase 4 | *hyfJ* | 2.1 | 9.9 | 11.0 | AEH-0001329 | YintA_01001346 |
| hydrogenase 3 and formate hydrogenase complex, HycG subunit/hydrogenase 4, Fe-S subunit | *hyfI* | 2.2 | 9.9 | 11.0 | AEH-0001330 | YintA_01001347 |
| hydrogenase 4, Fe-S subunit | *hyfH* | 2.2 | 9.9 | 11.0 | AEH-0001331 | YintA_01001348 |
| hydrogenase 3, large subunit/ hydrogenase 4, subunit | *hyfG* | 2.1 | 10.1 | 11.2 | AEH-0001332 | YintA_01001349 |
| hydrogenase 4, membrane subunit | *hyfF* | 1.8 | 10.3 | 11.2 | AEH-0001333 | YintA_01001350 |
| hydrogenase 3, membrane subunit | *hycD* | 1.9 | 9.9 | 10.9 | AEH-0001336 | YintA_01001353 |
| protein involved in nickel insertion into hydrogenases 3 | *hypA* | 1.9 | 9.8 | 10.8 | AEH-0001339 | YintA_01001357 |
| *menaquinone* |  |  |  |  |  |  |
| dihydroxynaphthoic acid synthetase | *menB* | 2.2 | 11.1 | 12.2 | AEH-0003549 | YintA_01003563 |
| 2-succinyl-6-hydroxy-2,4-cyclohexadiene-1-carboxylate synthase | *menD* | 1.9 | 10.0 | 11.0 | AEH-0003547 | YintA_01003561 |
| 2-succinyl-5-enolpyruvyl-6-hydroxy-3-cyclohexene-1-carboxylate synthase | *menD* | 1.7 | 10.1 | 10.9 | AEH-0003546 | YintA_01003560 |
| (1R,6R)-2-succinyl-6-hydroxy-2,4-cyclohexadiene-1-carboxylate synthase | *menH* | 1.8 | 10.7 | 11.5 | AEH-0003548 | YintA_01003562 |
| o-succinylbenzoyl-CoA synthase | *menC* | 2.0 | 10.1 | 11.1 | AEH-0003550 | YintA_01003564 |
| *anaerobically functioning peptidases* |  |  |  |  |  |  |
| predicted peptidase (collagenase-like) | *yhbU* | 4.2 | 9.9 | 11.9 | AEH-0001615 | YintA_01001639 |
| predicted protease | *yhbV* | 2.7 | 10.5 | 11.9 | AEH-0001616 | YintA_01001640 |
| peptidase T | *pepT* | 4.3 | 10.0 | 12.1 | AEH-0000070 | YintA_01000069 |
| *TCA* |  |  |  |  |  |  |
| lipoamide dehydrogenase, E3 component is part of three enzyme complexes | *lpd* | -2.2 | 14.1 | 13.0 | AEH-0002901 | YintA_01002924 |
| pyruvate dehydrogenase, dihydrolipoyltransacetylase component E2 | *aceF* | -2.3 | 13.5 | 12.2 | AEH-0002902 | YintA_01002925 |
| pyruvate dehydrogenase, decarboxylase component E1, thiamin-binding | *aceE* | -2.6 | 14.7 | 13.3 | AEH-0002903 | YintA_01002926 |
| DNA-binding transcriptional dual regulator | *pdhR* | -2.5 | 12.7 | 11.3 | AEH-0002904 | YintA_01002927 |
| bifunctional aconitate hydratase 2/2-methylisocitrate dehydratase | *acnB* | -1.7 | 12.7 | 11.9 | AEH-0002898 | YintA_01002921 |
| isocitrate dehydrogenase, specific for NADP+ | *icd* | -2.5 | 14.3 | 13.0 | AEH-0000080 | YintA_01000079 |
| succinate dehydrogenase, membrane subunit, binds cytochrome b556 | *sdhD* | -3.2 | 12.9 | 11.2 | AEH-0002154 | YintA_01002182 |
| succinate dehydrogenase, flavoprotein subunit | *sdhA* | -3.1 | 12.8 | 11.1 | AEH-0002155 | YintA_01002183 |
| 2-oxoglutarate decarboxylase, thiamin-requiring | *sucA* | -1.8 | 13.5 | 12.6 | AEH-0002157 | YintA_01002185 |
| succinate dehydrogenase iron-sulfur protein | *sdhB* | -2.6 | 12.1 | 10.7 | AEH-0002156 | YintA_01002184 |
| succinate dehydrogenase, membrane subunit, binds cytochrome b556 | *sdhC* | -3.5 | 13.8 | 12.0 | AEH-0002153 | YintA_01002181 |
| lipoyl-protein ligase | *lipB* | -1.9 | 13.4 | 12.5 | AEH-0001186 | YintA_01001201 |
| lipoate synthase | *lipA* | -1.9 | 13.1 | 12.1 | AEH-0001185 | YintA_01001200 |
| malate dehydrogenase, NAD(P)-binding | *mdh* | -2.6 | 13.5 | 12.1 | AEH-0002567 | YintA_01002592 |
| malate synthase A | *aceB* | -2.7 | 11.9 | 10.5 | AEH-0003794 | YintA_01003797 |
| isocitrate lyase | *aceA* | -2.2 | 11.6 | 10.5 | AEH-0003795 | YintA_01003798 |
| fumarate hydratase (fumarase C),aerobic Class II | *fumC* | -2.0 | 13.1 | 12.1 | AEH-0000609 | YintA_01000613 |
| *nucleotide metabolism* |  |  |  |  |  |  |
| glutaredoxin-like protein | *nrdH* | -14.2 | 13.5 | 9.7 | AEH-0002348 | YintA_01002375 |
| ribonucleoside-diphosphate reductase 2, beta subunit, ferritin-like | *nrdF* | -11.6 | 14.1 | 10.6 | AEH-0002345 | YintA_01002372 |
| protein that stimulates ribonucleotide reduction | *nrdI* | -13.8 | 14.4 | 10.6 | AEH-0002347 | YintA_01002374 |
| ribonucleoside-diphosphate reductase 2, alpha subunit | *nrdE* | -11.6 | 13.8 | 10.3 | AEH-0002346 | YintA_01002373 |
| anaerobic ribonucleoside-triphosphate reductase | *nrdD* | 4.4 | 10.8 | 12.9 | AEH-0001596 | YintA_01001620 |
| *cytochrome related* |  |  |  |  |  |  |
| cytochrome o ubiquinol oxidase subunit II | *cyoA* | -3.1 | 13.5 | 11.9 | AEH-0000872 | YintA_01000885 |
| cytochrome o ubiquinol oxidase subunit IV | *cyoD* | -3.1 | 14.6 | 13.0 | AEH-0000875 | YintA_01000888 |
| protoheme IX farnesyltransferase | *cyoE* | -2.3 | 12.6 | 11.4 | AEH-0000876 | YintA_01000889 |
| predicted cytochrome b561 | *yceJ* | -1.9 | 10.4 | 9.5 | AEH-0001069 | YintA_01001082 |
| cytochrome o ubiquinol oxidase subunit III | *cyoC* | -3.4 | 14.3 | 12.5 | AEH-0000874 | YintA_01000887 |
| cytochrome o ubiquinol oxidase subunit I | *cyoB* | -3.3 | 14.9 | 13.2 | AEH-0000873 | YintA_01000886 |
| *Fe-S Cluster Assembly* |  |  |  |  |  |  |
| ATP synthase, membrane-bound accesory subunit | *atpI* | 1.8 | 12.2 | 13.0 | AEH-0002237 | YintA_01002265 |
| Fe-S cluster assembly protein | *sufA* | -2.8 | 12.8 | 11.3 | AEH-0000223 | YintA_01000226 |
| component of SufBCD complex | *sufB* | -2.4 | 11.9 | 10.7 | AEH-0000224 | YintA_01000227 |
| transport protein associated with Fe-S cluster assembly | *sufC* | -2.6 | 13.7 | 12.3 | AEH-0000225 | YintA_01000228 |
| COG0719: ABC-type transport system involved in Fe-S cluster assembly, permease component | *sufD_N* | -2.2 | 12.5 | 11.3 | AEH-0000226 | YintA_01000229 |
| component of SufBCD complex | *sufD_C* | -2.0 | 12.2 | 11.1 | AEH-0000754 | YintA_01000762 |
| selenocysteine lyase, PLP-dependent | *sufS* | -1.9 | 11.8 | 10.9 | AEH-0000753 | YintA_01000761 |
| Fe-S cluster-containing transcription factor | *iscR* | -3.6 | 13.8 | 12.0 | AEH-0003496 | YintA_01003511 |
| *Glycolysis* |  |  |  |  |  |  |
| pyruvate kinase I | *pykF* | 2.0 | 13.6 | 14.7 | AEH-0000749 | YintA_01000756 |
| pyruvate kinase II | *pykA* | 2.3 | 12.3 | 13.5 | AEH-0000230 | YintA_01000232 |
| enolase | *eno* | 2.2 | 13.2 | 14.3 | AEH-0000474 | YintA_01000477 |
| glucosephosphate isomerase | *pgi* | 1.9 | 12.3 | 13.3 | AEH-0003061 | YintA_01003087 |
| triosephosphate isomerase | *tpiA* | 2.1 | 13.3 | 14.3 | AEH-0002796 | YintA_01002821 |
| phosphoglycero mutase III, cofactor-independent | *gpmM* | 3.6 | 13.0 | 14.8 | AEH-0002780 | YintA_01002805 |
| phosphoglyceromutase 1 | *gpmA* | -2.0 | 14.6 | 13.6 | AEH-0003618 | YintA_01003631 |
| phosphoglycerate kinase | *pgk* | 2.2 | 13.5 | 14.7 | AEH-0003856 | YintA_01003855 |
| phosphoenolpyruvate carboxylase | *ppc* | 1.8 | 12.8 | 13.7 | AEH-0003911 | YintA_01003908 |
|  |  |  |  |  |  |  |
| **2. cofactor metabolism** |  |  |  |  |  |  |
| precorrin-4 C11-methyltransferase | *cbiF* | 3.3 | 10.9 | 12.6 | AEH-0002872 | YintA_01002896 |
| cobyric acid synthase | *cbiP* | 1.7 | 11.1 | 11.9 | AEH-0002882 | YintA_01002906 |
| cobalamin 5'-phosphate synthase | *cobS* | 1.7 | 10.4 | 11.2 | AEH-0002884 | YintA_01002908 |
| putative cobalt transport protein | *cbiQ* | 2.1 | 11.0 | 12.1 | AEH-0002880 | YintA_01002904 |
| synthesis of vitamin B12 adenosyl cobalamide precursor | *cbiN* | 2.3 | 11.5 | 12.7 | AEH-0002879^e^ | YintA_01002903 |
| putative membrane protein | *cbiM* | 2.3 | 12.1 | 13.3 | AEH-0002878 | YintA_01002902 |
| precorrin-2 C20-methyltransferase | *cbiL* | 2.6 | 10.3 | 11.6 | AEH-0002877 | YintA_01002901 |
| cobalt chelatase | *cbiK* | 2.7 | 10.4 | 11.8 | AEH-0002876 | YintA_01002900 |
| precorrin-6X reductase | *cbiJ* | 3.0 | 11.1 | 12.7 | AEH-0002875 | YintA_01002899 |
| precorrin-8X methylmutase | *cbiC* | 2.0 | 10.9 | 11.9 | AEH-0002868 | YintA_01002892 |
| conserved hypothetical protein | *cbiG* | 2.9 | 10.6 | 12.1 | AEH-0002873 | YintA_01002897 |
| cobyrinic acid A,C-diamide synthase | *cbiA* | 1.9 | 9.7 | 10.6 | AEH-0002866 | YintA_01002890 |
| precorrin-8W decarboxylase | *cbiT* | 2.1 | 9.4 | 10.5 | AEH-0002871 | YintA_01002895 |
| precorrin-6Y C5,15-methyltransferase [decarboxylating] | *cbiE* | 2.2 | 9.6 | 10.8 | AEH-0002870 | YintA_01002894 |
| conserved hypothetical protein | *cbiD* | 2.1 | 10.3 | 11.4 | AEH-0002869 | YintA_01002893 |
| putative cobalt transport ATP-binding protein | *cbiO* | 2.0 | 10.6 | 11.6 | AEH-0002881 | YintA_01002905 |
| precorrin-3 C17-methyltransferase | *cbiH* | 2.8 | 10.2 | 11.7 | AEH-0002874 | YintA_01002898 |
| putative L-threonine-O-3-phosphate decarboxylase | *cobD* | 2.8 | 9.7 | 11.2 | AEH-0001382 | YintA_01001401 |
| pdu and cob operons regulatory protein | *pocR* | 1.7 | 10.1 | 10.8 | AEH-0002865 | YintA_01002888 |
| GTP cyclohydrolase I | *folE* | -2.0 | 12.1 | 11.2 | AEH-0001318 | YintA_01001334 |
| thiamin phosphate synthase (thiamin phosphate pyrophosphorylase) | *thiE* | -1.7 | 13.3 | 12.6 | AEH-0002409 | YintA_01002437 |
| thiamin (pyrimidine moiety) biosynthesis protein | *thiC* | -2.0 | 14.7 | 13.7 | AEH-0002410 | YintA_01002438 |
| Histidinol-phosphate/aromatic aminotransferase and cobyric acid decarboxylase | | 2.8 | 9.8 | 11.2 | AEH-0002842 | YintA_01002865 |
|  |  |  |  |  |  |  |
| **3. Carbohydrate Transport and Metabolism** |  |  |  |  |  |  |
| glucokinase | *glk* | 2.1 | 11.8 | 12.9 | AEH-0001103 | YintA_01001118 |
| glycerol kinase | *glpK* | 1.8 | 10.7 | 11.5 | AEH-0002803 | YintA_01002828 |
| PTS system glucose-specific IICB component | *ptsG* | 2.3 | 13.3 | 14.5 | AEH-0000054 | YintA_01000053 |
| IIA and HPr components of fructose-specific PTS enzyme | *fruB* | 2.4 | 10.8 | 12.0 | AEH-0003827 | YintA_01003827 |
| fructose-1-phosphate kinase | *fruK* | 2.0 | 11.6 | 12.7 | AEH-0003828 | YintA_01003828 |
| PTS system fructose-specific IIB'BC component | *fruA* | 1.8 | 11.5 | 12.4 | AEH-0003829 | YintA_01003829 |
| glucitol/sorbitol-specific enzyme IIC component of PTS | *srlA* | 3.3 | 11.1 | 12.8 | AEH-0002540 | YintA_01002566 |
| glucitol/sorbitol-specific enzyme IIB component of PTS | *srlE* | 2.9 | 10.3 | 11.8 | AEH-0002541 | YintA_01002567 |
| glucitol/sorbitol-specific enzyme IIA component of PTS | *srlB* | 3.3 | 9.8 | 11.5 | AEH-0002542 | YintA_01002568 |
| sorbitol-6-phosphate dehydrogenase | *srlD* | 2.5 | 11.8 | 13.1 | AEH-0002543 | YintA_01002569 |
| mannose-specific enzyme IID component of PTS | *manZ* | 2.2 | 12.2 | 13.3 | AEH-0000109 | YintA_01000108 |
| PTS system mannose-specific IIAB component | *manX* | 2.0 | 13.8 | 14.8 | AEH-0000111 | YintA_01000110 |
| putative membrane protein |  | 1.8 | 11.6 | 12.5 | AEH-0003210 | YintA_01003235 |
| phosphotransferase enzyme II, B component |  | 2.6 | 11.0 | 12.4 | AEH-0003211 | YintA_01003236 |
| phosphotransferase enzyme II, A component |  | 2.0 | 10.5 | 11.5 | AEH-0003212 | YintA_01003237 |
| ATP-dependent transcriptional activator of the maltose regulon | *malT* | 1.9 | 10.5 | 11.5 | AEH-0001539 | YintA_01001560 |
| L-glutamine:D-fructose-6-phosphate aminotransferase | *glmS* | 1.9 | 12.8 | 13.7 | AEH-0002247 | YintA_01002275 |
| fructose-6-phosphate aldolase 2 | *mipB* | 1.7 | 10.8 | 11.6 | AEH-0002735 | YintA_01002761 |
| 3-deoxy-D-arabino-heptulosonate-7-phosphate synthase, tyrosine-repressible | *aroF* | 1.8 | 12.7 | 13.5 | AEH-0003730 | YintA_01003737 |
| bifunctional heptose 7-phosphate kinase/heptose 1-phosphate adenyltransferase | *rfaE* | 1.8 | 12.0 | 12.8 | AEH-0003784 | YintA_01003787 |
| sugar phosphatase | *yfbT* | 2.5 | 11.2 | 12.5 | AEH-0003880 | YintA_01003879 |
| predicted transporter | *yfbS* | 1.7 | 11.3 | 12.0 | AEH-0003881 | YintA_01003880 |
| ADP-ribose diphosphatase | *nudE* | -1.8 | 11.8 | 11.0 | AEH-0003758 | YintA_01003763 |
|  |  |  |  |  |  |  |
| **4. Amino acid Transport and Metabolism** |  |  |  |  |  |  |
| *Other amino acids* |  |  |  |  |  |  |
| glutamate decarboxylase | *gadA* | -4.3 | 12.8 | 10.7 | AEH-0000007 | YintA_01000006 |
| predicted glutamate:gamma-aminobutyric acid antiporter | *gadC* | -4.1 | 12.2 | 10.1 | AEH-0000008 | YintA_01000007 |
| predicted glutaminase | *ybaS* | -3.4 | 12.7 | 10.9 | AEH-0000009 | YintA_01000008 |
| aminomethyltransferase, tetrahydrofolate-dependent, subunit (T protein) of glycine cleavage complex | *gcvT* | -2.3 | 12.6 | 11.4 | AEH-0001228 | YintA_01001243 |
| glycine cleavage complex lipoylprotein | *gcvH* | -2.3 | 14.1 | 12.9 | AEH-0001229 | YintA_01001244 |
| glycine decarboxylase, PLP-dependent, subunit (protein P) of glycine cleavage complex | *gcvP* | -1.8 | 12.3 | 11.5 | AEH-0001230 | YintA_01001245 |
| alanine racemase 2, PLP-binding | *dadX* | -3.0 | 11.6 | 10.0 | AEH-0002484 | YintA_01002510 |
| D-amino acid dehydrogenase | *dadA* | -5.5 | 13.1 | 10.6 | AEH-0002485 | YintA_01002511 |
| diaminopimelate decarboxylase, PLP-binding | *lysA* | -2.2 | 12.3 | 11.2 | AEH-0001272 | YintA_01001288 |
| dihydrodipicolinate reductase | *dapB* | -2.2 | 13.1 | 12.0 | AEH-0003534 | YintA_01003548 |
| L-allo-threonine dehydrogenase, NAD(P)-binding | *ydfG* | 2.2 | 11.2 | 12.3 | AEH-0000621 | YintA_01000625 |
| periplasmic L-asparaginase II | *ansB* | 2.5 | 9.7 | 11.0 | AEH-0000974 | YintA_01000987 |
| aspartate ammonia-lyase | *aspA* | 1.8 | 10.6 | 11.4 | AEH-0003031 | YintA_01003055 |
| 3'-phosphoadenosine 5'-phosphosulfate reductase | *cysH* | 1.8 | 13.0 | 13.9 | AEH-0000480 | YintA_01000483 |
| cysteine synthase A, O-acetylserine sulfhydrolase A subunit | *cysK* | 2.0 | 13.2 | 14.2 | AEH-0001117 | YintA_01001132 |
| *Methionine* |  |  |  |  |  |  |
| predicted C-N hydrolase family amidase, NAD(P)-binding | *yafV* | -2.3 | 12.3 | 11.1 | AEH-0001801 | YintA_01001824 |
| methionine aminotransferase, PLP-dependent | *ybdL* | -3.7 | 13.7 | 11.8 | AEH-0001802 | YintA_01001825 |
| probable dehydratase | *mtnB* | -2.6 | 14.0 | 12.6 | AEH-0001803 | YintA_01001826 |
| bifunctional enolase-phosphatase | *mtnC* | -2.5 | 15.0 | 13.6 | AEH-0001804 | YintA_01001827 |
| dioxygenase | *mtnD* | -2.1 | 13.8 | 12.7 | AEH-0001805 | YintA_01001828 |
| probable aldose-ketose isomerase | *mtnA* | -3.8 | 12.8 | 10.8 | AEH-0001806 | YintA_01001829 |
| probable 5-methylthioribose kinase | *mtnK* | -14.5 | 15.0 | 11.1 | AEH-0001807 | YintA_01001830 |
| methionine sulfoxide reductase B | *msrB* | -2.1 | 12.2 | 11.2 | AEH-0002475 | YintA_01002501 |
| homocysteine-N5-methyltetrahydrofolate transmethylase, B12-dependent | *metH* | -2.3 | 13.8 | 12.6 | AEH-0003066 | YintA_01003092 |
| 5-methyltetrahydropteroyltriglutamate-homocysteine S-methyltransferase | *metE* | -8.9 | 15.9 | 12.7 | AEH-0001651 | YintA_01001674 |
| ATP-binding component of DL-methionine uptake transporter (MUT) | *metN* | -2.0 | 12.9 | 12.0 | AEH-0002413 | YintA_01002440 |
| membrane component of DL-methionine uptake transporter (MUT) | *metI* | -2.5 | 14.7 | 13.4 | AEH-0002414 | YintA_01002441 |
| homoserine transsuccinylase | *metA* | -4.8 | 14.1 | 11.8 | AEH-0003793 | YintA_01003796 |
| 5,10-methylenetetrahydrofolate reductase | *metF* | -4.2 | 15.1 | 13.1 | AEH-0003912 | YintA_01003909 |
| bifunctional aspartokinase/homoserine dehydrogenase | *metL* | -2.7 | 13.3 | 11.9 | AEH-0003913 | YintA_01003910 |
| cystathionine gamma-synthase, PLP-dependent | *metB* | -3.6 | 15.3 | 13.5 | AEH-0003914 | YintA_01003911 |
| DNA-binding transcriptional activator, homocysteine-binding | *metR* | -3.3 | 12.5 | 10.8 | AEH-0004039 |  |
| CP4-6 prophage; S-methylmethionine:homocysteine methyltransferase | *mmuM* | -2.7 | 12.8 | 11.4 | AEH-0003449^s^ | YintA_01003465 |
| CP4-6 prophage; predicted S-methylmethionine transporter | *mmuP* | -2.0 | 12.3 | 11.3 | AEH-0003450^e^ | YintA_01003466 |
| *Amino acid and peptide transport* |  |  |  |  |  |  |
| cysteine and O-acetylserine exporter | *eamB* | -2.0 | 13.2 | 12.2 | AEH-0001938 | YintA_01001962 |
| sodium:serine/threonine symporter | *sstT* | 2.0 | 12.3 | 13.3 | AEH-0003672 | YintA_01003682 |
| predicted serine transporter | *sdaC* | 2.1 | 10.6 | 11.7 | AEH-0003322 | YintA_01003343 |
| periplasmic dipeptide transport protein | *dppA* | 2.3 | 13.7 | 14.9 | AEH-0003976 | YintA_01003962 |
| dipeptide transporter; ATP-binding component of ABC superfamily | *dppF* | 2.5 | 12.6 | 13.9 | AEH-0003653 | YintA_01003664 |
| dipeptide transporter; ATP-binding component of ABC superfamily | *dppD* | 2.8 | 12.3 | 13.8 | AEH-0003654 | YintA_01003665 |
| dipeptide transporter; membrane component of ABC superfamily | *dppC* | 3.2 | 12.0 | 13.6 | AEH-0003655 | YintA_01003666 |
| dipeptide transporter; membrane component of ABC superfamily | *dppB* | 3.5 | 12.0 | 13.8 | AEH-0003656 | YintA_01003667 |
| proton-dependent peptide transporter | *tppB* | 2.8 | 11.0 | 12.5 | AEH-0000718 | YintA_01000724 |
|  |  |  |  |  |  |  |
| **5. Stress-related** |  |  |  |  |  |  |
| *Oxidative stress* |  |  |  |  |  |  |
| lipid hydroperoxide peroxidase | *tpx* | -2.1 | 12.4 | 11.3 | AEH-0000699 | YintA_01000705 |
| monothiol glutaredoxin | *grxD* | -1.9 | 12.3 | 11.4 | AEH-0000738 | YintA_01000744 |
| superoxide dismutase, Fe | *sodB* | 6.8 | 11.5 | 14.3 | AEH-0000740 | YintA_01000746 |
| superoxide dismutase, Mn | *sodA* | -5.0 | 13.1 | 10.8 | AEH-0002108 | YintA_01002134 |
| bifunctional nitric oxide dioxygenase/dihydropteridine reductase 2 | *hmp* | -1.7 | 10.8 | 10.0 | AEH-0003504 | YintA_01003519 |
| catalase | *katA* | 2.4 | 10.4 | 11.6 | AEH-0003552 | YintA_01003566 |
| *General stress* |  |  |  |  |  |  |
| predicted universal stress protein A | *uspA* | 2.9 | 11.3 | 12.9 | AEH-0001502 | YintA_01001522 |
| predicted universal stress protein B | *uspB* | -2.2 | 12.6 | 11.5 | AEH-0001503 | YintA_01001523 |
| SOS cell division inhibitor | *sulA* | -2.9 | 12.8 | 11.3 | AEH-0000931 | YintA_01000944 |
| soluble pyridine nucleotide transhydrogenase | *sthA* | -2.5 | 12.9 | 11.6 | AEH-0003359 | YintA_01003378 |
| *starvation* |  |  |  |  |  |  |
| carbon starvation protein | *cstA* | -7.2 | 14.2 | 11.3 | AEH-0001647 | YintA_01001670 |
| carbon starvation protein | *cstA* | -2.1 | 10.9 | 9.8 | AEH-0002737 | YintA_01002763 |
| *osmotic stress* |  |  |  |  |  |  |
| predicted periplasmic-binding component of an ABC superfamily transporter | *osmF* | -1.9 | 12.9 | 12.0 | AEH-0002938 | YintA_01002960 |
| *acid stress* |  |  |  |  |  |  |
| COG0288: Carbonic anhydrase |  | -2.5 | 13.4 | 12.1 | AEH-0001262 | YintA_01001277 |
| COG3338: Carbonic anhydrase |  | 2.2 | 9.9 | 11.1 | AEH-0003890^e^ | YintA_01003888 |
|  |  |  |  |  |  |  |
| **6. Taxis, Motility, Attachment** |  |  |  |  |  |  |
| *Flagellum* |  |  |  |  |  |  |
| flagellar protein potentiates polymerization | *fliS* | 3.2 | 12.2 | 13.9 | AEH-0000403 | YintA_01000406 |
| predicted chaperone | *fliT* | 3.0 | 11.5 | 13.0 | AEH-0000404 | YintA_01000407 |
| flagellar biosynthesis protein | *fliL* | 2.3 | 11.2 | 12.4 | AEH-0000415 | YintA_01000418 |
| flagellar hook-length control protein | *fliK* | 2.2 | 10.5 | 11.6 | AEH-0000414 | YintA_01000417 |
| flagellar basal-body MS-ring and collar protein | *fliF* | 2.0 | 10.5 | 11.5 | AEH-0000409 | YintA_01000412 |
| flagellar biosynthesis protein | *fliH* | 2.2 | 10.5 | 11.7 | AEH-0000411 | YintA_01000414 |
| flagellar protein | *fliJ* | 1.9 | 11.4 | 12.3 | AEH-0000413 | YintA_01000416 |
| flagellum-specific ATP synthase | *fliI* | 1.8 | 10.4 | 11.2 | AEH-0000412 | YintA_01000415 |
| flagellar motor switching and energizing component | *fliG* | 2.1 | 10.9 | 12.0 | AEH-0000410 | YintA_01000413 |
| flagellar basal-body component | *fliE* | 2.4 | 12.1 | 13.3 | AEH-0000408 | YintA_01000411 |
| flagellar hook protein | *flgE* | 2.9 | 12.3 | 13.8 | AEH-0000429 | YintA_01000432 |
| flagellar filament structural protein (flagellin) | *fliC* | 2.2 | 14.4 | 15.5 | AEH-0000401^e^ | YintA_01000404 |
| RNA polymerase, sigma 28 (sigma F) factor | *fliA* | 2.6 | 11.2 | 12.6 | AEH-0000400 | YintA_01000403 |
| export chaperone for FlgK and FlgL | *flgN* | 1.7 | 11.0 | 11.7 | AEH-0000435 | YintA_01000438 |
| predicted regulator of FliA activity | *fliZ* | 2.4 | 12.0 | 13.3 | AEH-0000399 | YintA_01000402 |
| flagellar filament capping protein | *fliD* | 3.0 | 12.3 | 13.9 | AEH-0000402 | YintA_01000405 |
| assembly protein for flagellar basal-body periplasmic P ring | *flgA* | 1.7 | 11.1 | 11.9 | AEH-0000433 | YintA_01000436 |
| flagellar component of cell-proximal portion of basal-body rod | *flgB* | 2.6 | 12.5 | 13.9 | AEH-0000432 | YintA_01000435 |
| flagellar component of cell-proximal portion of basal-body rod | *flgC* | 2.7 | 12.4 | 13.9 | AEH-0000431 | YintA_01000434 |
| flagellar biosynthesis protein | *fliO* | 2.1 | 10.6 | 11.7 | AEH-0000418 | YintA_01000421 |
| flagellar hook assembly protein | *flgD* | 3.2 | 11.1 | 12.8 | AEH-0000430 | YintA_01000433 |
| flagellar motor switching and energizing component | *fliM* | 1.8 | 11.4 | 12.3 | AEH-0000416 | YintA_01000419 |
| flagellar component of cell-proximal portion of basal-body rod | *flgF* | 3.4 | 11.1 | 12.8 | AEH-0000428 | YintA_01000431 |
| flagellar component of cell-distal portion of basal-body rod | *flgG* | 3.0 | 11.5 | 13.1 | AEH-0000427 | YintA_01000430 |
| flagellar protein of basal-body outer-membrane L ring | *flgH* | 2.1 | 10.9 | 12.0 | AEH-0000426 | YintA_01000429 |
| predicted flagellar basal body protein | *flgI* | 2.0 | 10.4 | 11.4 | AEH-0000425 | YintA_01000428 |
| muramidase | *flgJ* | 2.1 | 10.9 | 12.0 | AEH-0000424 | YintA_01000427 |
| flagellar hook-filament junction protein 1 | *flgK* | 3.6 | 11.4 | 13.3 | AEH-0000423 | YintA_01000426 |
| flagellar hook-filament junction protein | *flgL* | 3.2 | 10.7 | 12.4 | AEH-0000422 | YintA_01000425 |
| DNA-binding transcriptional dual regulator with FlhC | *flhD* | 2.2 | 10.0 | 11.1 | AEH-0003279 | YintA_01003302 |
| DNA-binding transcriptional dual regulator with FlhD | *flhC* | 1.9 | 10.7 | 11.7 | AEH-0003280 | YintA_01003303 |
| chemotaxis MotA protein | *motA* | 2.9 | 11.2 | 12.7 | AEH-0003281 | YintA_01003304 |
| chemotaxis MotB protein | *motB* | 2.2 | 10.8 | 11.9 | AEH-0003282 | YintA_01003305 |
| *Taxis* |  |  |  |  |  |  |
| aerotaxis signal transducer | *aer* | 3.8 | 10.1 | 12.0 | AEH-0000085 | YintA_01000084 |
| chemotaxis regulator, protein phosphatase for CheY | *cheZ* | 2.5 | 11.2 | 12.6 | AEH-0000440 | YintA_01000443 |
| chemotaxis regulator transmitting signal to flagellar motor component | *cheY* | 2.3 | 12.7 | 13.9 | AEH-0000441 | YintA_01000444 |
| fused chemotaxis regulator and protein-glutamate methylesterase in two-component regulatory system with CheA | *cheB* | 2.1 | 12.0 | 13.1 | AEH-0000442 | YintA_01000445 |
| chemotaxis regulator, protein-glutamate methyltransferase | *cheR* | 2.2 | 10.7 | 11.8 | AEH-0000443 | YintA_01000446 |
| methyl-accepting protein IV | *tap* | 2.3 | 11.2 | 12.4 | AEH-0000444 | YintA_01000001 |
| methyl-accepting chemotaxis protein II | *cheM* | 3.1 | 12.2 | 13.9 | AEH-0002107 | YintA_01002133 |
| Chemotaxis signal transduction protein | *cheV* | 2.3 | 10.9 | 12.1 | AEH-0002676 | YintA_01002702 |
| methyl-accepting chemotaxis protein III, ribose and galactose sensor receptor | *trg* | 3.4 | 10.3 | 12.1 | AEH-0003274 | YintA_01003297 |
| chemotaxis protein CheA | *cheA* | 2.9 | 11.1 | 12.6 | AEH-0003283 | YintA_01003306 |
| purine-binding chemotaxis protein | *cheW* | 2.9 | 11.2 | 12.7 | AEH-0003284 | YintA_01003307 |
| methyl-accepting chemotaxis protein | *tar* | 2.7 | 12.2 | 13.6 | AEH-0003285 | YintA_01003308 |
| *Adhesion, Attachment* |  |  |  |  |  |  |
| predicted fimbrial-like adhesin protein | *yfcV* | -1.8 | 11.2 | 10.4 | AEH-0000518 | YintA_01000522 |
| COG3539: P pilus assembly protein, pilin FimA |  | 2.7 | 12.1 | 13.5 | AEH-0000354^e^ | YintA_01000356 |
| P pilus assembly/Cpx signaling pathway, periplasmic inhibitor/zinc- resistance associated protein | *cpxP* | 2.1 | 10.6 | 11.7 | AEH-0002790 | YintA_01002815 |
| DNA-binding transcriptional regulator | *crl* | -1.8 | 12.8 | 11.9 | AEH-0001829 | YintA_01001852 |
|  |  |  |  |  |  |  |
| **7. urea metabolism** |  |  |  |  |  |  |
| urease gamma subunit | *ureA* | -2.3 | 13.5 | 12.2 | AEH-0001784 | YintA_01001808 |
| urease beta subunit | *ureB* | -2.0 | 12.0 | 11.0 | AEH-0001783 | YintA_01001807 |
| urease alpha subunit | *ureC* | -2.2 | 13.3 | 12.2 | AEH-0001782 | YintA_01001805 |
| urease accessory protein | *ureE* | -2.0 | 12.8 | 11.8 | AEH-0001781 | YintA_01001804 |
| urease accessory protein | *ureF* | -1.8 | 12.3 | 11.4 | AEH-0001780 | YintA_01001803 |
| ureidoglycolate hydrolase | *allA* | 1.8 | 10.8 | 11.6 | AEH-0003291^s^ | YintA_01003313 |
|  |  |  |  |  |  |  |
| **8. Iron Metabolism** |  |  |  |  |  |  |
| transducer of proton motive force for active transport | *tonB* | -2.3 | 11.3 | 10.1 | AEH-0002374 | YintA_01002401 |
| putative iron transport permease |  | -4.1 | 13.3 | 11.3 | AEH-0002762 | YintA_01002787 |
| iron-enterobactin outer membrane transporter | *fepA* | -4.4 | 12.3 | 10.1 | AEH-0002899 | YintA_01002922 |
| putative iron transport protein |  | -6.5 | 13.1 | 10.4 | AEH-0002763 | YintA_01002788 |
| putative iron transport permease |  | -2.7 | 12.0 | 10.6 | AEH-0002761 | YintA_01002786 |
| putative iron ABC transporter, ATP-binding protein |  | -2.5 | 11.6 | 10.3 | AEH-0002760 | YintA_01002785 |
| putative ferric siderophore receptor | *iutA* | -23.0 | 15.0 | 10.5 | AEH-0002759 | YintA_01002784 |
| putative siderophore biosynthesis protein IucD | *iucD* | -26.6 | 14.7 | 10.0 | AEH-0002758 | YintA_01002783 |
| putative siderophore biosynthetic enzyme | *ysuG* | -11.8 | 13.4 | 9.8 | AEH-0002757 | YintA_01002782 |
| putative siderophore biosynthesis protein IucB | *iucB* | -13.5 | 13.5 | 9.7 | AEH-0002756 | YintA_01002781 |
|  |  | -20.7 | 14.2 | 9.9 | AEH-0002755 | YintA_01002780 |
| ferrichrome outer membrane transporter | *fhuA* | -6.5 | 13.4 | 10.7 | AEH-0002044 | YintA_01002069 |
| iron(III)-transport system permease | *sfuB* | -1.8 | 11.3 | 10.4 | AEH-0001083 | YintA_01001096 |
| iron(III)-binding periplasmic protein | *sfuA* | -5.5 | 12.8 | 10.4 | AEH-0001082 | YintA_01001095 |
| ATP-binding component of iron-hydroxamate transporter | *fhuC* | -2.0 | 11.0 | 10.0 | AEH-0000458 | YintA_01000461 |
| ferrichrome outer membrane transporter | *fhuA* | -10.3 | 13.7 | 10.3 | AEH-0001869^e^ | YintA_01001892 |
| membrane spanning protein in TonB-ExbB-ExbD complex | *exbD* | -3.3 | 12.1 | 10.3 | AEH-0001883 | YintA_01001906 |
| enterobactin/ferric enterobactin esterase | *fes* | -4.4 | 12.0 | 9.9 | AEH-0001868 | YintA_01001891 |
| periplasmic-binding component of an ABC superfamily iron-enterobactin transporter | *fepB* | -2.3 | 11.3 | 10.0 | AEH-0001864 | YintA_01001887 |
| Fe-binding and storage protein | *dps* | -1.8 | 12.1 | 11.3 | AEH-0001300 | YintA_01001316 |
| bacterioferritin-associated ferredoxin | *bfd* | -5.1 | 13.3 | 10.9 | AEH-0001961 | YintA_01001985 |
| outer membrane transporter for monomeric catechol-containing ferric ion-siderophore complexes | *yiuR* | -25.8 | 15.0 | 10.3 | AEH-0003834 | YintA_01003834 |
| ATP-binding component of ABC superfamily iron-enterobactin transporter | *fepC* | -1.9 | 10.8 | 9.9 | AEH-0001867 | YintA_01001890 |
| conserved protein | *yfeX* | 2.7 | 11.9 | 13.4 | AEH-0003161 | YintA_01003187 |
| ferritin iron storage protein (cytoplasmic) | *ftnA* | -2.2 | 11.5 | 10.4 | AEH-0000126 | YintA_01000125 |
| ferrous iron permease | *efeU* | -4.1 | 12.0 | 9.9 | AEH-0000391 | YintA_01000394 |
| redox component of a tripartite ferrous iron transporter | *efeB* | -2.9 | 12.1 | 10.6 | AEH-0000389 | YintA_01000392 |
| component of a tripartite ferrous iron transporter | *efeO* | -4.5 | 12.1 | 9.9 | AEH-0000390 | YintA_01000393 |
| hemin-binding periplasmic protein | *hemT* | -7.3 | 13.0 | 10.1 | AEH-0002048 | YintA_01002074 |
| hemin transport system permease protein HmuU | *hemU* | -5.0 | 12.7 | 10.4 | AEH-0002047 | YintA_01002073 |
| hemin transport protein | *hemS* | -13.9 | 15.1 | 11.3 | AEH-0002049 | YintA_01002075 |
| hemin receptor precursor | *hemR* | -26.6 | 14.9 | 10.1 | AEH-0002050 | YintA_01002076 |
| hemin uptake protein | *hemP* | -37.7 | 15.2 | 10.0 | AEH-0002051 | YintA_01002077 |
| hemin transport system ATP-binding protein | *hemV* | -3.3 | 11.6 | 9.9 | AEH-0002046 | YintA_01002072 |
| membrane spanning protein in TonB-ExbB-ExbD complex | *exbB* | -3.8 | 12.6 | 10.7 | AEH-0001884 | YintA_01001907 |
| chelated iron transport system membrane protein | *yfeD* | -2.0 | 12.6 | 11.6 | AEH-0000175 | YintA_01000176 |
| ATP-binding transport protein | *yfeB* | -2.5 | 12.9 | 11.6 | AEH-0000177 | YintA_01000178 |
| chelated iron transport system membrane protein | *yfeC* | -2.1 | 12.3 | 11.3 | AEH-0000176 | YintA_01000177 |
| periplasmic-binding protein | *yfeA* | -6.1 | 13.8 | 11.2 | AEH-0000178 | YintA_01000179 |
| periplasmic protein p19 involved in high-affinity Fe2+ transport | | -1.8 | 10.6 | 9.8 | AEH-0000320 | YintA_01000322 |
|  |  |  |  |  |  |  |
| **9. Secretion/Export** |  |  |  |  |  |  |
| ssrAB activated gene | *srfA* | 1.9 | 11.3 | 12.2 | AEH-0000648 | YintA_01000654 |
| ssrAB activated gene | *srfB* | 2.0 | 11.0 | 12.0 | AEH-0000649 | YintA_01000655 |
| ssrAB activated gene: predicted coiled-coil structure | *srfC* | 2.0 | 11.1 | 12.2 | AEH-0000650 | YintA_01000656 |
| secreted protein | *yceI* | -2.0 | 10.7 | 9.7 | AEH-0001068 | YintA_01001081 |
| RTX toxins and related Ca2+-binding proteins |  | -1.9 | 11.0 | 10.1 | AEH-0001136^e^ | YintA_01001150 |
| RTX toxins and related Ca2+-binding proteins |  | -3.6 | 12.9 | 11.1 | AEH-0001137^e^ | YintA_01001151 |
| hypothetical protein |  | -2.6 | 12.0 | 10.6 | AEH-0001138^e^ | YintA_01001152 |
| ABC-type protease/lipase transport system, ATPase and permease components | | -1.7 | 11.1 | 10.3 | AEH-0001139 | YintA_01001153 |
| component of Sec-independent translocase | *tatE* | 2.1 | 11.4 | 12.4 | AEH-0001184 | YintA_01001199 |
| outer membrane protein W | *ompW* | 3.6 | 9.9 | 11.7 | AEH-0002368 | YintA_01002395 |
| outer membrane porin protein C | *ompC* | 2.0 | 11.0 | 12.0 | AEH-0003226 | YintA_01003250 |
|  |  |  |  |  |  |  |
| **10. Other** |  |  |  |  |  |  |
| RNA polymerase, sigma S (sigma 38) factor | *rpoS* | -2.5 | 12.6 | 11.3 | AEH-0000499 | YintA_01000503 |
| regulator of sigma D | *rsd* | -1.9 | 11.6 | 10.7 | AEH-0003918 | YintA_01003914 |
| recombination and repair protein | *recN* | -2.0 | 12.0 | 11.0 | AEH-0001747 | YintA_01001770 |
| DNA-damage-inducible protein I | *dinI* | -1.8 | 11.3 | 10.4 | AEH-0000035 | YintA_01000034 |
| DNA polymerase IV | *dinB* | -1.9 | 12.2 | 11.3 | AEH-0001825 | YintA_01001848 |
| DNA polymerase V, subunit D | *umuD* | -1.9 | 11.1 | 10.2 | AEH-0000088 | YintA_01000087 |
| uridine phosphorylase | *udp* | -1.8 | 12.9 | 12.1 | AEH-0001649 | YintA_01001672 |
| uracil transporter | *uraA* | 2.1 | 12.7 | 13.8 | AEH-0002518 | YintA_01002543 |
| uracil phosphoribosyltransferase | *upp* | 2.0 | 12.8 | 13.8 | AEH-0002519 | YintA_01002544 |
| c-di-GMP binding protein | *ycgR* | 1.9 | 12.0 | 12.9 | AEH-0001835 | YintA_01001858 |
| 3-oxoacyl-[acyl-carrier-protein] synthase I | *fabB* | 2.0 | 13.2 | 14.2 | AEH-0001030 | YintA_01001042 |
| long-chain fatty acid outer membrane transporter | *fadL* | 2.0 | 11.3 | 12.3 | AEH-0001043 | YintA_01001055 |
| FKBP-type peptidyl-prolyl cis-trans isomerase (rotamase) | *fklB* | 1.8 | 11.3 | 12.1 | AEH-0002583 | YintA_01002608 |
| Elongation factor EF-P | *efp* | 2.0 | 11.7 | 12.8 | AEH-0003037 | YintA_01003061 |
| sulfate transporter subunit; periplasmic-binding component of ABC superfamily | *sbp* | 1.9 | 11.7 | 12.6 | AEH-0002795 | YintA_01002820 |
|  |  |  |  |  |  |  |
| **11. Uncharacterized proteins** |  |  |  |  |  |  |
| putative membrane protein |  | -2.0 | 10.5 | 9.5 | AEH-0001350 | YintA_01001368 |
| conserved protein | *ydiY* | 1.7 | 10.7 | 11.4 | AEH-0003727 | YintA_01003734 |
| conserved protein | *yigI* | -1.9 | 10.6 | 9.7 | AEH-0001696 | YintA_01001720 |
| conserved protein | *ygiW* | -1.8 | 12.4 | 11.6 | AEH-0003163 | YintA_01003189 |
| conserved protein | *yfbU* | 2.2 | 12.5 | 13.7 | AEH-0003879 | YintA_01003878 |
| conserved hypothetical protein |  | 2.3 | 10.7 | 11.9 | AEH-0000349 | YintA_01000351 |
| AraC-family transcriptional regulator |  | -4.3 | 12.3 | 10.2 | AEH-0002900 | YintA_01002923 |
| predicted transcriptional regulator | *ychQ* | 1.7 | 10.6 | 11.4 | AEH-0000268 | YintA_01000270 |
| putative exported protein |  | -4.8 | 12.4 | 10.1 | AEH-0002497 | YintA_01002523 |
| conserved inner membrane protein associated with acetate transport | *yaaH* | 2.0 | 12.1 | 13.1 | AEH-0003247 | YintA_01003270 |
| predicted protein | *yjjZ* | -3.3 | 12.9 | 11.2 | AEH-0002173 | YintA_01002201 |
| Putative transport protein |  | -5.6 | 13.6 | 11.1 | AEH-0002088 | YintA_01002114 |
| putative membrane transport protein |  | -1.8 | 12.2 | 11.4 | AEH-0003448 | YintA_01003464 |
| putative exported protein |  | -1.8 | 11.0 | 10.2 | AEH-0001559^e^ | YintA_01001582 |
| Putative threonine efflux protein |  | 1.9 | 12.1 | 13.0 | AEH-0003531 | YintA_01003545 |
| predicted arginine/ornithine antiporter transporter | *ydgI* | -1.7 | 12.3 | 11.5 | AEH-0002193 | YintA_01002222 |
| putative membrane protein |  | -1.7 | 11.1 | 10.3 | AEH-0000319 | YintA_01000321 |
| ABC transporter, ATP-binding protein |  | -1.8 | 12.1 | 11.2 | AEH-0000316 | YintA_01000318 |
| predicted transporter | *ybhI* | 2.7 | 10.4 | 11.8 | AEH-0001447 | YintA_01001467 |
| predicted sulphate anion transporter | *ychM* | 2.1 | 12.0 | 13.1 | AEH-0000265 | YintA_01000267 |
| putative sugar ABC transporter |  | -3.6 | 14.2 | 12.3 | AEH-0001349 | YintA_01001367 |
| conserved hypothetical protein |  | -2.3 | 13.1 | 11.9 | AEH-0002089 | YintA_01002115 |
| predicted DNA-binding transcriptional regulator | *yidP* | 1.7 | 10.2 | 11.0 | AEH-0002071^e^ | YintA_01002097 |
| predicted DNA-binding transcriptional regulator | *yheO* | 1.7 | 11.8 | 12.6 | AEH-0001953 | YintA_01001977 |
| putative membrane receptor protein |  | -1.7 | 11.6 | 10.8 | AEH-0001925 | YintA_01001949 |
| conserved hypothetical protein |  | 1.7 | 10.2 | 11.0 | AEH-0000182 | YintA_01000183 |
| predicted peptidase | *yegQ* | 2.5 | 10.6 | 11.9 | AEH-0002538 | YintA_01002564 |
| conserved hypothetical protein |  | -3.2 | 13.6 | 11.9 | AEH-0001347 | YintA_01001365 |
| N-terminal fragment of a hypothetical protein (pseudogene) |  | -3.2 | 14.1 | 12.4 | AEH-0001348 | YintA_01001366 |
| predicted Fe-S oxidoreductase | *yhcC* | 2.6 | 11.2 | 12.5 | AEH-0003103 | YintA_01003128 |
| predicted oxidoreductase | *ydhF* | 1.7 | 11.5 | 12.3 | AEH-0000731 | YintA_01000737 |
| Arylsulfatase regulator (Fe-S oxidoreductase) |  | 1.7 | 11.4 | 12.1 | AEH-0001058^s^ | YintA_01001071 |
| predicted oxidoreductase | *ydjA* | -2.5 | 13.4 | 12.1 | AEH-0002470 | YintA_01002496 |
| radical SAM protein | *ygiQ* | 1.8 | 11.1 | 11.9 | AEH-0001890 | YintA_01001913 |
| predicted oxidoreductase | *aegA* | 4.6 | 10.3 | 12.5 | AEH-0003023 | YintA_01003047 |
| predicted FAD-linked oxidoreductase | *ydiJ* | 1.7 | 12.4 | 13.1 | AEH-0000221 | YintA_01000224 |
| predicted oxidoreductase, Zn-dependent and NAD(P)-binding | *yhdH* | 2.7 | 10.6 | 12.0 | AEH-0001433 | YintA_01001453 |
| putative peptidase | *pepP1* | -3.4 | 13.3 | 11.5 | AEH-0000310 | YintA_01000312 |
| putative methyl-accepting chemotaxis protein | *tsr2* | 3.2 | 10.5 | 12.2 | AEH-0003641 | YintA_01003653 |
| Putative methyl-accepting chemotaxis receptor protein |  | 1.9 | 10.6 | 11.5 | AEH-0003560 | YintA_01003574 |
| Methyl-accepting chemotaxis protein |  | 4.7 | 10.1 | 12.3 | AEH-0000378^e^ | YintA_01000381 |
| Methyl-accepting chemotaxis protein |  | 4.2 | 10.7 | 12.8 | AEH-0003898 | YintA_01003897 |
| conserved inner membrane protein | *yeaQ* | -2.8 | 13.4 | 11.9 | AEH-0002924 | YintA_01002946 |
| hypothetical protein |  | -21.4 | 15.0 | 10.6 | AEH-0000311 | YintA_01000313 |
| predicted hydrolase | *ysgA* | -1.8 | 12.1 | 11.3 | AEH-0001650 | YintA_01001673 |
| putative exported protein |  | 2.1 | 11.1 | 12.2 | AEH-0001112 | YintA_01001127 |
| putative exported protein |  | 1.8 | 11.0 | 11.8 | AEH-0002769 | YintA_01002795 |
| hypothetical protein |  | 6.8 | 10.8 | 13.5 | AEH-0003219^e^ | YintA_01003243 |
| hypothetical protein |  | -3.1 | 12.2 | 10.6 | AEH-0002349^e^ | YintA_01002376 |
| hypothetical protein |  | 1.8 | 13.3 | 14.2 | AEH-0004003^e^ | YintA_01003975 |
| conserved protein | *ycbJ* | 2.3 | 11.1 | 12.3 | AEH-0000960 | YintA_01000973 |
| hypothetical protein |  | 2.0 | 10.7 | 11.7 | AEH-0000181 | YintA_01000182 |
| putative membrane protein |  | -1.7 | 11.6 | 10.8 | AEH-0000612 | YintA_01000616 |
| predicted protein | *yejG* | -4.1 | 11.8 | 9.8 | AEH-0003478 | YintA_01003494 |
| hypothetical protein |  | 4.6 | 10.0 | 12.2 | AEH-0000514^s^ | YintA_01000518 |
| putative starvation-inducible protein | *psiF* | -1.8 | 10.9 | 10.1 | AEH-0003617 | YintA_01003630 |
| conserved hypothetical protein |  | -2.3 | 11.1 | 9.9 | AEH-0002727 | YintA_01002753 |
| predicted inner membrane protein | *yjjP* | 3.2 | 11.2 | 12.8 | AEH-0003530 | YintA_01003544 |
| Putative inner membrane protein |  | -1.7 | 12.3 | 11.5 | AEH-0001409 | YintA_01001429 |
| predicted inner membrane protein | *yigM* | -1.9 | 12.4 | 11.5 | AEH-0001652 | YintA_01001676 |
| conserved inner membrane protein | *yeiB* | -1.8 | 11.8 | 11.0 | AEH-0001319 | YintA_01001335 |
| conserved inner membrane protein | *yjjB* | 2.2 | 11.5 | 12.6 | AEH-0003529 | YintA_01003543 |
| putative inner membrane protein |  | -1.9 | 12.1 | 11.1 | AEH-0001461^e^ | YintA_01001482 |
| predicted siderophore interacting protein | *yqjH* | -2.6 | 11.1 | 9.7 | AEH-0000302 | YintA_01000304 |
| predicted lipid carrier protein | *yhbT* | 1.7 | 10.9 | 11.7 | AEH-0001614 | YintA_01001638 |
| putative Phospholipid-binding protein | *ybhB* | -1.8 | 12.8 | 12.0 | AEH-0000386^e^ | YintA_01000389 |
| predicted acetyltransferase | *yhhY* | 1.9 | 11.3 | 12.3 | AEH-0001519 | YintA_01001539 |
| NADP-dependent alcohol dehydrogenase | *yqhD* | 1.8 | 10.6 | 11.4 | AEH-0001888 | YintA_01001911 |
| putative ABC transport protein, ATP-binding component |  | -1.8 | 12.0 | 11.1 | AEH-0001560^e^ | YintA_01001583 |
| conserved protein | *yobA* | -1.9 | 11.6 | 10.7 | AEH-0000127 | YintA_01000126 |
| predicted esterase | *yeiG* | 1.7 | 11.2 | 12.0 | AEH-0001314 | YintA_01001330 |
| conserved protein | *ycfP* | 1.7 | 11.7 | 12.5 | AEH-0000061 | YintA_01000060 |
| Putative threonine efflux protein |  | -2.3 | 12.2 | 11.0 | AEH-0002752^s^ | YintA_01002777 |
| conserved protein | *yfcZ* | 3.5 | 11.6 | 13.4 | AEH-0001042 | YintA_01001054 |
| Siderophore-interacting protein |  | -3.5 | 12.1 | 10.3 | AEH-0002753^e^ | YintA_01002778 |
| conserved protein | *yeaD* | 2.3 | 12.4 | 13.6 | AEH-0002477 | YintA_01002503 |
| conserved protein | *ycgL* | -1.9 | 12.1 | 11.1 | AEH-0002496 | YintA_01002522 |
| conserved protein | *ycgB* | -1.9 | 12.0 | 11.1 | AEH-0002486 | YintA_01002512 |
| conserved protein with nucleoside triphosphate hydrolase domain | *yeaG* | -1.9 | 12.8 | 11.8 | AEH-0002479 | YintA_01002505 |
| putative membrane protein |  | -2.2 | 11.7 | 10.6 | AEH-0002754 | YintA_01002779 |
| putative inner membrane protein |  | -1.9 | 11.1 | 10.2 | AEH-0002350 | YintA_01002377 |
| conserved protein | *yeaH* | -1.9 | 11.9 | 11.0 | AEH-0002480 | YintA_01002506 |
| conserved hypothetical protein |  | 1.9 | 10.0 | 10.9 | AEH-0001304 | YintA_01001320 |
| predicted transporter | *yhjX* | -2.7 | 11.9 | 10.5 | AEH-0001102 | YintA_01001117 |
| conserved hypothetical protein |  | 3.3 | 9.9 | 11.6 | AEH-0001174 | YintA_01001188 |
|  |  |  |  |  |  |  |
| **12. Non protein coding RNAs** |  |  |  |  |  |  |
| regulatory antisense RNA | *ryhB* | -5.4 | 12.2 | 9.8 | AEH-0004004 |  |
| hypothetical regulatory antisense RNA | *ryhB2* | -4.4 | 11.8 | 9.7 | AEH-0004011 |  |
|  | *fnrS* | 8.9 | 9.9 | 13.0 | AEH-0004014 |  |
| tRNA-Leu(CAA) |  | -1.8 | 11.9 | 11.0 | AEH-0004072 |  |

**Table S1**
